# The therapeutic potential of botanicals: how medicinal plants targeting autophagy can reverse metabolic-associated fatty liver disease

**DOI:** 10.3389/fphar.2026.1776868

**Published:** 2026-04-07

**Authors:** Kuisong Wang, Bing Jiang, Xupeng Huang, Yuqi Zhang, Haotian Qi, Shengxian Xu, Li Sun, Yanjing Liu

**Affiliations:** 1 College of Traditional Chinese Medicine, Changchun University of Chinese Medicine, Changchun, Jilin, China; 2 Department of Infection, The First Affiliated Hospital of Army Medical University, Chongqing, China; 3 Acupuncture and Tuina Academy, Changchun University of Chinese Medicine, Changchun, Jilin, China; 4 Department of Geriatrics, The First Clinical Hospital of Jilin Academy of Traditional Chinese Medicine, Changchun, Jilin, China; 5 Department of Endocrinology, Metabolism and Gastroenterology, Third Affiliated Clinical Hospital to Changchun University of Chinese Medicine, Changchun, Jilin, China

**Keywords:** autophagy pathway, bioactive compounds, medicinal plant extracts, metabolic-associated fatty liver disease, natural herbal plants, traditional Chinese medicine compound preparations

## Abstract

Metabolic-associated fatty liver disease (MAFLD) is a serious condition that can progress to cirrhosis and liver cancer. Natural herbal therapeutics, characterized by their multi-constituent and multi-target properties, as well as favorable safety profiles—particularly lower hepatorenal toxicity—are attracting significant research interest for MAFLD management. In this review, we examine their ethnopharmacological applications, with a focus on autophagy regulation. Information was gathered from traditional medical texts and online databases (e.g., PubMed and CNKI) using keywords such as “MAFLD,” “autophagy,” and “natural herbal plants.” Incorporating herbal plants into MAFLD treatment offers several advantages. First, autophagy regulation involves multiple signaling pathways (e.g., PI3K/AKT/mTOR, AMPK/TFEB, PINK1/Parkin, and Unc-51-like autophagy activating kinase 1 (ULK1)/Beclin-1/VPS34). Single-target drugs often fail to modulate this complex network effectively, whereas various medicinal plants and their bioactive compounds can simultaneously interact with key targets such as mTOR, AMPK, TFEB, SIRT1, LC3B, Beclin-1, ATG5, ULK1, and PPARγ. Second, these plants demonstrate excellent safety profiles. Traditional Chinese compound preparations, such as Zexie Decoction and Shenling Baizhu Powder, have shown clinical efficacy over centuries. To elucidate their mechanisms, researchers are now isolating bioactive compounds from these formulas for cellular and animal studies, revealing their specific roles in modulating autophagy. In summary, plant-derived bioactive compounds—especially those targeting autophagy—have shown promising clinical results against MAFLD and represent valuable candidates for future drug development.

## 1 Introduction

The nomenclature for metabolic fatty liver disease has evolved in parallel with advances in disease definition. The international community has proposed the terms metabolic dysfunction-associated steatotic liver disease (MASLD) and its inflammatory subtype, metabolic dysfunction-associated steatohepatitis (MASH), as updated designations intended to encompass the disease spectrum previously described by non-alcoholic fatty liver disease (NAFLD) and, in part, metabolic-associated fatty liver disease (MAFLD). In this review, we use MAFLD as the umbrella term for fatty liver disease associated with metabolic dysfunction and MASH to denote the steatohepatitis phenotype. Importantly, differences in diagnostic criteria across these definitions may affect comparability among studies, cohorts, and regions. MAFLD is a highly prevalent chronic liver disorder closely associated with insulin resistance (IR) and systemic metabolic dysfunction. The disease encompasses hepatic steatosis, inflammation, and progressive fibrosis and can advance from simple steatosis to MASH, advanced fibrosis, cirrhosis, and hepatocellular carcinoma (HCC) (Badmus et al., 2022). Driven by the increasing prevalence of obesity, type 2 diabetes (T2D), and metabolic syndrome (MetS), MAFLD has become a leading cause of chronic liver disease (CLD) worldwide, with a rapidly increasing burden in China (Wong et al., 2021; Venkatesan and Haroon, 2023). No pharmacotherapies are currently approved specifically for MAFLD. Therefore, clinical management remains focused on lifestyle modifications and the control of metabolic comorbidities. Although glucagon-like peptide-1 receptor agonists (GLP-1RAs) and peroxisome proliferator-activated receptor (PPAR) agonists can benefit certain subsets of patients at a higher risk of disease progression, most available interventions primarily slow, rather than reverse, hepatic inflammation and fibrosis. This underscores the need for more effective and individualized treatment strategies (Habibullah et al., 2024).

Against this backdrop, traditional Chinese medicine (TCM), often conceptualized as a multifaceted approach, has been used as an adjunctive therapy for MAFLD (Wen et al., 2025). Although MAFLD is not a formal diagnostic entity within TCM nosology, its clinical manifestations are commonly correlated with related syndrome patterns to guide individualized treatment prescriptions. Mechanistic studies suggest that certain natural products and isolated compounds can enhance lipid metabolism and reduce inflammation and fibrosis through pathways including PPARα/INSIG/SREBP1c and the transforming growth factor-β (TGF-β)/Smad/mitogen-activated protein kinase (MAPK) signaling pathway (Gu et al., 2022; Wu et al., 2019). Consistently, clinical studies report improvements in IR, aminotransferase levels, steatosis, and fibrosis-related indices when certain formulas are combined with lifestyle interventions (Zhou et al., 2025; Jin et al., 2024).

Hepatocellular lipid overload is a key driver of MAFLD, with excess lipid accumulation promoting oxidative stress, endoplasmic reticulum (ER) stress, inflammation, and fibrogenesis (Bae et al., 2022). Autophagy (particularly lipophagy), along with mitophagy and ER-phagy, counteracts these insults by clearing excess lipid droplets (LDs) and damaged organelles, thereby helping maintain metabolic homeostasis (Chen and Lin, 2022; Zhang et al., 2022). Autophagy impairment promotes lipid droplet accumulation and amplifies oxidative stress, ER stress, inflammation, and fibrosis. Conversely, MAFLD-related IR can further suppress autophagy, creating a self-reinforcing pathogenic loop (Zhao et al., 2021; Chen and Lin, 2022). Accordingly, restoring autophagic flux (by enhancing lipophagy or mitophagy) represents a promising therapeutic strategy.

In this review, we summarize the autophagy pathways and regulatory mechanisms involved in the progression of MAFLD and synthesize evidence that natural products and herbal bioactives may ameliorate steatosis, inflammation, and fibrosis by modulating autophagy, thereby supporting their potential as candidate interventions for MAFLD.

## 2 Literature search and methods

This study systematically reviews *in vitro*, *in vivo*, and clinical evidence on the protective effects and molecular mechanisms of medicinal plants in MAFLD, with a focus on autophagy-related signaling pathways. We searched PubMed, CNKI, Web of Science, and Wanfang Database from inception to December 2025 using terms related to MAFLD (including “MAFLD” OR “MASLD” OR “NAFLD” OR “MASH” OR “NASH” OR “fatty liver” OR “hepatic steatosis”), autophagy (including “autophagy” OR “macroautophagy” OR “mitophagy” OR “lipophagy”), and natural products (including “medicinal plants” OR “TCM” OR “plant extracts” OR “bioactive compounds”). Reference lists of relevant articles were manually screened. We included original research (*in vitro*, *in vivo*, or clinical) and high-quality reviews investigating medicinal plants in MAFLD-related liver injury or fibrosis, with mechanistic data involving autophagy. We excluded unpublished manuscripts, conference abstracts, case reports, commentaries, and studies lacking mechanistic investigations. Only articles published in English or Chinese were considered. After deduplication and preliminary screening, 692 articles were identified. Two independent reviewers screened the titles, abstracts, and full texts, resolving disagreements through discussion or consultation with a third reviewer. Ultimately, 235 studies were included. Data extraction included medicinal plant and compound names, experimental models, key findings, and autophagy-related mechanisms. Due to heterogeneity in interventions and outcomes, a narrative synthesis was performed. All plant species were taxonomically validated using the MPNS portal (http://mpns.kew.org/mpns-portal/), and complete scientific names were provided.

## 3 Overview of autophagy and its biological significance

Autophagy is a conserved, lysosome-dependent catabolic pathway, named from the Greek term for “self-eating,” that removes aberrant proteins, damaged organelles (e.g., mitochondria and ER), and invading pathogens. The resulting metabolites, including amino acids (AAs) and fatty acids (FAs), are recycled to support biosynthesis and ATP production, making autophagy essential for stress adaptation (e.g., starvation), proteostasis, and organelle quality control (Liu et al., 2023). Autophagic flux is tightly regulated and context-dependent, coordinated by autophagy-related genes (ATGs) within integrated signaling networks, where the AMPK/mTOR axis serves as a dominant initiation node.

**FIGURE 1:**
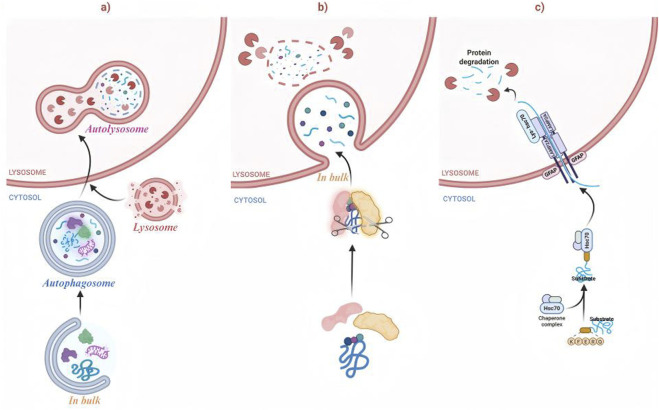
Autophagy encompasses three different forms, including macroautophagy, microautophagy, and CMA. CMA, chaperone-mediated autophagy. **(a)** Macroautophagy **(b)** Microautophagy **(c)** Chaperone-mediated autophagy (CMA).

Autophagy is a functional double-edged sword: basal activity is broadly cytoprotective, whereas insufficient or excessive autophagy can contribute to pathology (Malik et al., 2024). In macroautophagy, stress signals (e.g., hypoxia or nutrient deprivation) activate the Unc-51-like autophagy activating kinase 1 (ULK1)–ATG13–FIP200 (RB1CC1)–ATG101 complex, which recruits and activates the class III PI3K/VPS34 (PIK3C3) complex at phagophore initiation sites to drive membrane nucleation. Subsequent elongation and closure are mediated by two ubiquitin-like conjugation cascades, ATG12–ATG5–ATG16L1 and LC3/ATG8 lipidation to phosphatidylethanolamine (PE), resulting in the formation of a double-membraned autophagosome that sequesters cargo. Autophagosomes then fuse with lysosomes to form autolysosomes, thereby facilitating hydrolase-mediated degradation and the release of recycled metabolites. Autophagy is commonly categorized by its cargo delivery route into macroautophagy, microautophagy, or chaperone-mediated autophagy (CMA). In CMA, proteins containing KFERQ-like motifs are recognized and translocated via heat shock cognate protein 70 (HSC70) (HSPA8) and lysosome-associated membrane protein 2A (LAMP2A) (Figure 1) (Yamamoto and Matsui, 2024). Consistent with its central homeostatic role, autophagy dysfunction is implicated in major disorders, including neurodegenerative diseases (Nixon and Rubinsztein, 2024), cancer (Debnath et al., 2023), immune dysregulation (Hua et al., 2019), and metabolic diseases (Tao and Xu, 2020; Qian et al., 2021), highlighting the therapeutic potential of autophagy-targeted modulation.

**FIGURE 2:**
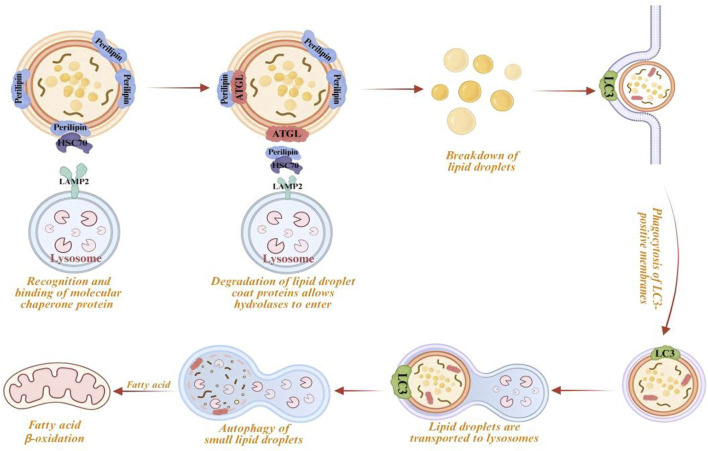
Occurrence process and molecular mechanism of lipophagy.

**FIGURE 3:**
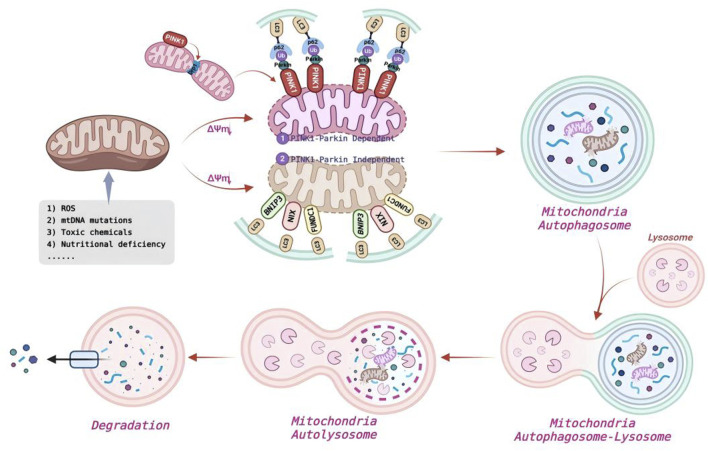
Occurrence process and molecular mechanism of mitophagy.

Among selective autophagy pathways, lipophagy and mitophagy play pivotal roles in lipid metabolism and mitochondrial quality control, respectively (Shin, 2020; Yang M. et al., 2024). Lipophagy mediates the lysosomal degradation of LDs (Figure 2), releasing FAs for β-oxidation and membrane biogenesis. In contrast, mitophagy removes damaged or excess mitochondria to limit reactive oxygen species (ROS) and maintain bioenergetic homeostasis (Figure 3). Both processes depend on the core ATG machinery, including ULK1, and are regulated by AMPK/mTOR signaling. However, they involve distinct upstream modules. Lipophagy is often enhanced by TFEB-driven transcriptional programs that increase lysosomal and autophagic capacity (Su et al., 2023), whereas mitophagy is typically mediated by the PINK1/PRKN (Parkin) pathway. Loss of mitochondrial membrane potential (ΔΨm) stabilizes PINK1 on the outer mitochondrial membrane (OMM), promotes PRKN-dependent ubiquitination of mitochondrial substrates, and recruits receptors such as OPTN and NDP52 (CALCOCO2) to link damaged mitochondria to LC3-positive membranes (Lin et al., 2024). Both pathways are dose-sensitive: appropriate lipophagy supports lipid homeostasis, whereas excessive activation may exacerbate lipotoxicity. Insufficient mitophagy allows the accumulation of dysfunctional mitochondria, while excessive clearance can compromise ATP supply (Dupont and Terzi, 2025). Mechanistically, lipophagy involves the recognition and remodeling of LD (e.g., PLIN2-associated modifications), engulfment by LC3-decorated membranes, and subsequent breakdown by lysosomal lipases (Corbo and Chung, 2024). Mitophagy depends on damage sensing, receptor-mediated sequestration, and lysosomal degradation (Lu et al., 2023). Dysregulated lipophagy is implicated in MAFLD, obesity, and IR, while impaired mitophagy contributes to neurodegenerative disorders and is also associated with diabetes, cardiomyopathy, aging, and cancer (Li W. et al., 2021). Notably, the concurrent failure of these pathways can establish a lipid–mitochondrial vicious cycle, in which lipid overload amplifies mitochondrial stress and decreasing mitochondrial function further limits FA oxidation, thereby accelerating metabolic deterioration (Talari et al., 2023). Defining the bidirectional crosstalk between lipophagy and mitophagy, along with disease-specific regulatory mechanisms, should reveal actionable targets for co-targeting selective autophagy in complex pathologies.

## 4 Regulatory mechanism and related signaling pathway of autophagy

### 4.1 Regulatory mechanisms of autophagy

#### 4.1.1 Regulation of nutrient perception and energy state

Cells regulate autophagy through an integrated network of nutrient and energy sensing, in which AMP-activated protein kinase (AMPK) and the mammalian target of rapamycin (mTOR) serve as key signaling hubs that detect changes in energy status and nutrient availability, respectively. AMPK is activated under conditions of energy deficiency, whereas mTOR is active when nutrients are abundant. These pathways interact antagonistically to maintain cellular metabolic homeostasis. During energy stress, an increased AMP/ATP ratio activates AMPK, which promotes autophagy through multiple mechanisms. First, AMPK phosphorylates and activates ULK1 to initiate autophagosome formation. Second, AMPK inhibits mTORC1 activity, thereby relieving its suppressive effect on autophagy. Additionally, AMPK can phosphorylate the transcription factor FOXO3, enhancing its nuclear translocation and promoting the transcription of ATGs (Kim et al., 2011). In contrast, nutrient sufficiency inhibits autophagy through mechanisms such as activation of mTORC1, inhibitory phosphorylation of ULK1, and cytoplasmic retention of TFEB (Nnah et al., 2019). Dysregulation of nutrient-sensing mechanisms is closely associated with the onset and progression of multiple diseases. In MetS, chronic nutrient excess leads to persistent activation of mTORC1, which impairs autophagy-mediated clearance of LD, thereby promoting hepatic lipid accumulation and the development of MAFLD (Zhang H. et al., 2018). In neurodegenerative diseases, impaired energy metabolism can disrupt AMPK signaling, while decreasing autophagic function promotes abnormal protein aggregation and accelerates neuronal degeneration (Ghavami et al., 2014). Within the tumor microenvironment, cancer cells remodel nutrient-sensing pathways to regulate autophagy in a manner that promotes survival. This adaptation allows them to utilize autophagy to withstand nutrient scarcity while preventing excessive autophagy when nutrients are abundant (Camuzard et al., 2020).

In recent years, targeting nutrient-sensing pathways has emerged as a promising therapeutic strategy. Caloric restriction and intermittent fasting enhance autophagy by cyclically activating AMPK and inhibiting mTOR, producing protective effects in models of metabolic and neurodegenerative diseases (Shabkhizan et al., 2023; Mattson et al., 2017). AMPK activators, such as metformin, can mimic an energy-stress state and restore autophagic function in metabolic disorders (Song et al., 2015). Additionally, natural flavonoids such as hesperidin have been reported to modulate autophagy-related processes in various cellular contexts, including supporting follicular development in 3D-cultured ovarian follicles (Shoorei et al., 2019), suggesting broader applications for autophagy modulation. Collectively, these intervention strategies underscore the therapeutic potential of modulating nutrient-sensing pathways and autophagy across a spectrum of diseases. However, their clinical translation faces critical challenges. A key consideration is that systemic activation of AMPK or inhibition of mTOR carries significant off-target risks, such as muscle wasting, highlighting the necessity of developing liver-selective delivery strategies (Li et al., 2023; Younossi et al., 2023). Moreover, the optimal therapeutic window for autophagy modulation in MAFLD remains poorly defined, necessitating careful dose optimization to balance therapeutic efficacy against the risk of maladaptive catabolic responses in extrahepatic tissues.

#### 4.1.2 Regulation of autophagy-related protein complexes

Autophagy-related protein complexes play central roles in the initiation, elongation, and maturation of autophagy, and their dysfunction is closely related to the onset and progression of various diseases. These complexes coordinate each stage of autophagy through the orderly assembly and disassembly of their components (Yao et al., 2024). The ULK1 complex (comprising ULK1, ATG13, FIP200, and ATG101), which serves as a key regulator of autophagy initiation, is activated by AMPK and inhibited by mTOR signaling. This regulation triggers autophagosome formation under conditions of nutrient deprivation. The complex phosphorylates downstream targets to recruit and activate the class III PI3K complex, thereby initiating the formation of the autophagosomal membrane. Dysfunction of the ULK1 complex has been associated with abnormal protein aggregation in neurodegenerative diseases, as well as with tumor initiation and progression (Lin and Hurley, 2016). Class III PI3K complexes, with Vps34 as the catalytic core subunit, generate phosphatidylinositol 3-phosphate (PI3P), a crucial lipid signal that recruits autophagy effector proteins containing FYVE or PX domains to the autophagic membrane (Ohashi et al., 2019). Beclin1 functions as a scaffold protein that dynamically regulates complex activity through interactions with multiple factors. Binding to UVRAG and ATG14L promotes autophagy, whereas binding to Rubicon inhibits this process. Dysregulation of this complex has been implicated in tumorigenesis, neurodegeneration, and pathogen infection (Boutouja et al., 2017). Two ubiquitin-like conjugation systems, namely, ATG12–ATG5–ATG16L1 and LC3–PE, collaboratively regulate autophagosome elongation and closure. The ATG12–ATG5–ATG16L1 complex functions as a molecular scaffold that modulates the curvature of the autophagic membrane, while lipidated LC3 facilitates membrane elongation and cargo recognition. Dysfunction in these systems disrupts autophagic flux and is associated with various metabolic and infectious diseases (Mooney and Sahingur, 2021). Finally, the HOPS complex-mediated autophagosome–lysosome fusion is a critical terminal step that coordinates membrane docking and fusion, enabling the efficient degradation of autophagic cargo. Impaired HOPS function has been associated with neurodegenerative diseases and lysosomal storage disorders (van der Beek et al., 2019).

Overall, these autophagy-related protein complexes maintain the dynamic balance of the autophagic process through precise and coordinated regulation. They are not only central to fundamental cell biology research but are also widely recognized as potential therapeutic targets for various diseases. Therefore, further elucidation of the regulatory networks involving these complexes and their mechanisms of action in specific disease contexts will provide a crucial theoretical foundation for developing novel autophagy-targeted therapies. A fundamental issue is that the stoichiometric balance between positive and negative regulators, such as Rubicon and Beclin1, ultimately determines autophagic output. However, the quantitative dynamics of these regulators in the human MAFLD liver remain largely uncharacterized. In addition, it is essential to map the cell-type-specific expression patterns of these complexes across hepatocytes, Kupffer cells, and stellate cells to accurately predict therapeutic selectivity. This is particularly important because current experimental approaches cannot reliably determine whether interventions would differentially affect lipid metabolism or inflammatory and fibrotic processes.

#### 4.1.3 Regulation of transcription factor levels

In the transcriptional regulatory network of autophagy, TFEB/TFE3 and the FOXO family, as two core transcription factors, collaboratively regulate autophagy–lysosomal function through distinct signaling pathways to maintain intracellular homeostasis (Lv et al., 2024). TFEB and TFE3 are key regulatory factors of the autophagy–lysosome pathway that enhance the cell’s degradative capacity by coordinating the transcriptional expression of ATGs and lysosomal genes. Under nutrient-rich conditions, mTORC1-mediated phosphorylation retains TFEB and TFE3 in the cytoplasm. However, during starvation or stress, they translocate to the nucleus, bind to CLEAR elements in the promoters of target genes, and activate the transcriptional programs of ATGs (such as LC3, ULK1, and p62) and lysosomal genes (such as cathepsins and LAMP1), thereby significantly enhancing the cell’s degradative capacity. This regulatory mechanism ensures that cells can precisely adjust autophagic activity in response to environmental changes (Pastore et al., 2017). The FOXO transcription factor family (including FOXO1, FOXO3, and others) plays a crucial role under conditions of energy and oxidative stress. These factors can directly bind to the promoter regions of ATGs, such as LC3, ULK1, and ATG12, while also engaging in cross-talk with core autophagy signaling pathways such as AMPK and mTOR. Under energy stress, FOXO and AMPK mutually activate each other and inhibit mTOR, forming a positive regulatory feedback loop. Additionally, FOXO3 helps maintain intracellular homeostasis by regulating the expression of mitophagy-related genes (PINK1 and BNIP3) and antioxidant genes (Orea-Soufi et al., 2022).

These two types of transcription factors exhibit both functional complementarity and network crosstalk in regulating autophagic activity. TFEB and TFE3 primarily govern the global regulation of the biogenesis and function of the autophagy–lysosome system, whereas FOXO is more specialized in activating autophagy and maintaining quality control under stress conditions. Their synergistic actions collectively ensure precise regulation of autophagy, and dysfunction in these factors is closely associated with various diseases. In neurodegenerative disorders, decreased activity of TFEB, TFE3, and FOXO may lead to impaired autophagic function and abnormal protein accumulation (Hu et al., 2019; Guan et al., 2024). In metabolic diseases, dysregulation disrupts lipid metabolism and impairs insulin sensitivity (Gros and Muller, 2023; Du and Zheng, 2021). During tumor development, these transcription factors may either inhibit tumor progression or support tumor cell survival in specific microenvironments, exhibiting complex, environment-dependent behavior (Farhan et al., 2017; Di Malta et al., 2023). Therefore, an in-depth analysis of the regulatory networks of various transcription factors in specific types of autophagy and disease contexts is crucial for developing novel therapeutic strategies targeting autophagy. These studies not only help elucidate the mechanisms underlying disease onset but also provide new molecular targets for precision medicine. A critical consideration arises from the observation that chronic TFEB activation may impose unsustainable metabolic demands on hepatocytes already overloaded with lipids. Moreover, the complex interplay between FOXO and TFEB, including their potential competition for shared transcriptional coactivators, remains entirely unexplored in the context of MAFLD. Addressing this knowledge gap will require generating stage-specific chromatin accessibility data to better predict cellular responses to transcription factor modulation and to prevent pushing cells beyond their metabolic capacity.

#### 4.1.4 Regulation of selective autophagy

Selective autophagy facilitates the targeted removal of damaged organelles, aberrant protein aggregates, and invading pathogens through specific autophagy receptors, thereby maintaining cellular homeostasis and enhancing stress resilience. Dysfunction of selective autophagy is closely associated with a variety of diseases (Wu et al., 2024). Mitophagy eliminates dysfunctional mitochondria via the PINK1/Parkin pathway and receptor-mediated mechanisms. The loss of mitochondrial membrane potential stabilizes PINK1, which subsequently recruits Parkin to ubiquitinate mitochondrial proteins. Autophagy receptors, such as OPTN and NDP52, then bind to LC3 and facilitate the delivery of damaged mitochondria for degradation (Eiyama and Okamoto, 2015). Under hypoxic conditions, receptors such as BNIP3 and NIX directly bind to LC3 to induce mitophagy (Zhang and Ney, 2009). Impaired mitophagy promotes the accumulation of damaged mitochondria and is implicated in diabetes, Parkinson’s disease, and ischemia–reperfusion (I/R) injury (Picca et al., 2023). ER-phagy identifies excessively expanded or damaged ER fragments through specific receptors such as FAM134B, RTN3L, and CCPG1. FAM134B, one of the first ER-phagy receptors to be identified, targets ER fragments for autophagic degradation via its LC3-interacting region (LIR). Mutations in FAM134B are directly associated with hereditary sensory neuropathy. Additionally, SEC62 mediates the autophagic clearance of fragmented ER during the recovery phase of ER stress (Mochida and Nakatogawa, 2022). Lipophagy regulates lipid metabolism by directly degrading LDs. Autophagosomes recognize proteins on the surface of LDs, such as PLIN2, or interact directly with the LDs, transporting them to lysosomes for degradation. This process releases free FAs for energy metabolism. Dysregulation of lipophagy is closely associated with metabolic diseases. In MAFLD, impaired lipophagy leads to abnormal accumulation of LDs within hepatocytes, exacerbating hepatic lipotoxicity (Minami et al., 2023). Aggrephagy of protein aggregates involves the recognition of ubiquitinated protein aggregates through receptors such as p62/SQSTM1 and NBR1. These receptors bind ubiquitinated proteins through their UBD domains and simultaneously interact with LC3 via their LIR domains, targeting abnormal proteins for autophagic degradation. In neurodegenerative diseases, dysfunction of this process leads to the accumulation of toxic proteins such as α-synuclein and tau, thereby accelerating disease progression (Simonsen and Wollert, 2022). Interestingly, different types of selective autophagy do not function independently but rather form a synergistic regulatory network. For example, defects in mitophagy lead to the accumulation of ROS, which can further exacerbate abnormal protein aggregation, while dysfunction in lipophagy disrupts lipid metabolic homeostasis. The synergistic interactions among these selective autophagy pathways collectively maintain the delicate balance within the cell.

Given the critical role of selective autophagy in various diseases, targeting specific selective autophagy pathways has emerged as a promising therapeutic strategy. For instance, enhancing mitophagy with small-molecule activators can alleviate pathological symptoms in models of neurodegenerative diseases, while modulating lipophagy shows potential as a treatment for metabolic disorders. Therefore, in-depth research into the specific regulatory mechanisms of various types of selective autophagy will provide a novel theoretical foundation for developing precise disease treatment strategies. A key point to recognize is that selective autophagy receptors function through phase-separation dynamics, a mechanism in which, paradoxically, high-affinity binding can sometimes hinder, rather than promote, progression. Under conditions of combined lipid and mitochondrial stress, competition between lipophagy and mitophagy for shared LC3 pools may ultimately determine which organelles are prioritized for clearance. Furthermore, the observation that autophagy deficiency promotes ferroptosis, while ferroptosis inhibition may impair lipid clearance, underscores the necessity of balanced co-targeting strategies rather than simply enhancing either pathway unidirectionally.

### 4.2 Autophagy-related signaling pathways

#### 4.2.1 PI3K/AKT/mTOR signaling pathway

The phosphoinositide 3-kinase (PI3K)/AKT/mTOR signaling pathway is a central cascade that regulates the autophagic process within cells. As a critical link between extracellular signals and intracellular autophagic activity, this pathway precisely controls the initiation and progression of autophagy by integrating signals such as growth factors, nutrient availability, and energy status. PI3K, once activated by growth factors such as insulin, catalyzes the production of PIP3, which subsequently recruits and activates AKT. Activated AKT inhibits the TSC1/TSC2 complex through phosphorylation, leading to the accumulation of Rheb-GTP and the activation of mTORC1, thereby suppressing autophagy (Tian et al., 2023). In the regulation of autophagy, the PI3K/AKT/mTOR signaling pathway primarily exerts its inhibitory effect through mTORC1. Activated mTORC1 directly phosphorylates ULK1 at the Ser757 site and ATG13 within the ULK1/ATG1 complex, suppressing their kinase activity and thereby blocking the initiation of autophagy. Additionally, mTORC1 phosphorylates the transcription factor TFEB, promoting its binding to 14-3-3 proteins and retention in the cytoplasm, which inhibits the transcriptional activation of autophagy- and lysosome-related genes (Roczniak-Ferguson et al., 2012). Notably, class I PI3K/AKT inhibits autophagy by activating mTORC1, whereas class III PI3K (Vps34) promotes autophagosome formation by forming complexes with proteins such as Beclin1. This highlights the complexity of this pathway in regulating autophagy (Cook et al., 2025). An increasing number of studies indicate that abnormal activation of this signaling pathway is closely associated with the onset and progression of various diseases. In tumors, persistent activation of the PI3K/AKT/mTOR signaling pathway inhibits autophagy, thereby promoting tumor cell proliferation and survival. Common mechanisms underlying this abnormal activation include PTEN loss and PIK3CA mutations (Yang et al., 2025). In neurodegenerative diseases, dysfunction of this pathway impairs autophagy’s ability to clear abnormal protein aggregates, thereby accelerating disease progression (Cai Y. et al., 2025). In metabolic diseases, IR results in impaired PI3K/AKT signaling and aberrant activation of mTORC1. These alterations collectively cause autophagic dysfunction, which promotes abnormal lipid accumulation and organ damage (Dong Y. et al., 2025).

Given the central role of the PI3K/AKT/mTOR pathway in regulating autophagy, intervention strategies targeting this pathway have become a crucial focus in disease treatment. For example, PI3K inhibitors (such as wortmannin), AKT inhibitors (such as capivasertib), and mTOR inhibitors (such as rapamycin) have demonstrated potential in preclinical studies to alleviate disease progression by restoring autophagic function (Lee et al., 2014). Particularly in the field of tumor treatment, combining PI3K/AKT/mTOR pathway inhibitors with conventional chemotherapy drugs may enhance the anti-tumor effect by regulating autophagy levels (Si et al., 2025). However, the specific functions of this pathway in different tissues and disease contexts require further elucidation to enable more precise targeted therapies. A significant therapeutic challenge arises from the opposing roles of class I and class III PI3Ks in autophagy regulation: although class I PI3K inhibits autophagy, class III PI3K promotes it. Consequently, non-selective PI3K inhibition may inadvertently suppress the pro-autophagic function of Vps34. In the context of MAFLD, the presence of hyperinsulinemia further complicates mTORC1 targeting, making it highly desirable to selectively disrupt the mTORC1–ULK1 interaction without globally inhibiting PI3K/AKT signaling. Additionally, the existence of a lipophagy–SREBP-1c feedback loop necessitates stage-specific targeting to avoid triggering compensatory lipogenesis.

#### 4.2.2 AMPK/ULK1 signaling pathway

AMPK is a highly conserved serine/threonine protein kinase that functions as a central sensor of cellular energy status, playing a crucial role in coordinating metabolic adaptation and autophagic regulation. AMPK typically exists as a heterotrimer composed of a catalytic α subunit and regulatory β and γ subunits, with its activity precisely regulated by the AMP/ATP ratio. Under conditions of cellular energy stress, the AMP/ATP ratio increases, activating AMPK through a dual mechanism involving allosteric activation and phosphorylation of the Thr172 site by upstream kinases such as LKB1 and CaMKKβ (Steinberg and Hardie, 2023). In the regulation of autophagy, AMPK promotes both the initiation and progression of the process through multiple mechanisms. First, AMPK directly phosphorylates several sites on the ULK1 complex, including Ser317 and Ser777, thereby enhancing its kinase activity. This activation leads to the phosphorylation of downstream autophagy-related proteins, initiating autophagosome formation. This mechanism counteracts the inhibitory effect of mTORC1 on ULK1, establishing a precise bidirectional regulatory system. Second, AMPK regulates the assembly of the core autophagy complex through phosphorylation, such as directly phosphorylating Beclin1 at Ser93 and Ser96, which promotes activation of the class III PI3K complex and facilitates nucleation of the autophagosome membrane. In addition, AMPK can indirectly promote autophagy by inhibiting mTORC1 activity. It enhances this inhibition by phosphorylating TSC2 and also directly phosphorylates a key component of mTORC1, RAPTOR, which induces its binding to 14-3-3 proteins, thereby inactivating mTORC1 (Kapuy et al., 2024). Notably, AMPK can also modulate the autophagic process by regulating the activity of transcription factors. It phosphorylates the transcription factor FOXO3, promoting its translocation to the nucleus and upregulating the expression of ATGs (Yang C. et al., 2020). Meanwhile, by activating the histone deacetylase SIRT1, it enhances both the activity and nuclear localization of the transcription factor TFEB, thereby comprehensively promoting the transcriptional expression of autophagy–lysosome genes (Huang et al., 2019). An increasing number of studies have found that dysfunction of the AMPK/ULK1 signaling axis is closely associated with the onset and progression of various diseases. In metabolic disorders, reduced AMPK activity impairs autophagy, exacerbating IR and hepatic lipid accumulation, thereby promoting the development of MAFLD (Lee et al., 2025). In neurodegenerative diseases, dysfunction of this pathway impairs the cell’s ability to clear abnormal protein aggregates, thereby accelerating disease progression (Mary et al., 2025). During tumorigenesis, the AMPK/ULK1 signaling pathway may exert tumor-suppressive effects by inhibiting mTOR and promoting autophagy. However, it can also support the survival of tumor cells under metabolic stress in specific microenvironments, demonstrating a complex, environment-dependent behavior (Zhao M. et al., 2024).

Given the central role of the AMPK/ULK1 signaling pathway in regulating autophagy, drug development targeting this pathway has become a crucial focus for disease treatment. AMPK activators, such as metformin, have demonstrated potential in treating metabolic diseases by enhancing autophagy to improve metabolic homeostasis (Duan et al., 2024). Additionally, natural compounds such as resveratrol can exert neuroprotective effects by activating the AMPK/ULK1 signaling pathway (Kang et al., 2020). Therefore, further investigation into the regulatory mechanisms of this signaling pathway under various disease conditions is essential to establish a robust theoretical foundation for developing precise, autophagy-targeted therapeutic strategies. It is important to note that AMPK exists as multiple heterotrimeric complexes with tissue-specific functions. However, current pharmacological activators lack hepatic selectivity, which poses a risk of undesirable effects in muscle tissue. Moreover, AMPK activation alone is unlikely to be sufficient in cases of impaired lysosomal acidification, a common feature of advanced MAFLD. Adding to the complexity, excessive autophagic induction may overwhelm the cell’s antioxidant capacity as ROS clearance by autophagy is intricately linked to Nrf2 signaling.

#### 4.2.3 Class III PI3K/Beclin1 complex pathway

The class III PI3K/Beclin1 complex is a core functional unit within the autophagy regulatory network, playing a pivotal role in the initiation and elongation of autophagosome formation (Ye et al., 2023). This complex, with Vps34 as its core catalytic subunit, generates PI3P, a key lipid messenger that provides essential signaling platforms for autophagic membrane assembly. The newly formed PI3P specifically recruits autophagy effector proteins containing FYVE or PX domains to the pre-autophagosomal structure, thereby initiating the nucleation process of the autophagosome. Beclin1, a crucial scaffold protein within the complex, not only maintains the structural stability and kinase activity of Vps34 but also dynamically integrates various regulatory factors through its specialized protein interaction domains (McKnight and Zhenyu, 2013). This dynamic assembly mechanism enables the complex to respond precisely to multiple intracellular signals. On one hand, positive regulatory factors such as Atg14L and UVRAG enhance the complex’s activity, promoting autophagosome formation. On the other hand, negative regulatory factors such as Rubicon restrict the autophagic process by inhibiting Vps34’s enzymatic activity and membrane localization. Furthermore, upstream signaling pathways such as AMPK promote autophagy by phosphorylating Beclin1, whereas Bcl-2 inhibits autophagic function under nutrient-rich conditions by binding to the BH3 domain of Beclin1 (Wang Y. et al., 2022). During autophagosome maturation, the spatiotemporal distribution of PI3P provides membrane anchor sites for key effector molecules, such as WIPI proteins and the ATG12–ATG5–ATG16L1 complex, which directly promote the elongation and eventual closure of the autophagic membrane (Gong et al., 2024; Shatz et al., 2024). The dysregulation of this specific regulatory mechanism is critically implicated in the onset and progression of several major diseases. During tumorigenesis, haploinsufficiency of Beclin1 impairs autophagic function, which, in turn, mediates genomic instability and drives tumor progression (Yue et al., 2003). In neurodegenerative diseases such as Alzheimer’s disease, the decrease in the function of this complex impairs the clearance of abnormal protein aggregates, thereby accelerating neuronal degeneration (Lachance et al., 2019). During pathogen infection, various microorganisms have evolved specific mechanisms to target this complex, thereby achieving immune evasion by interfering with autophagy (Cui et al., 2024).

The class III PI3K/Beclin1 complex plays a central role in the regulation of autophagy, making it a promising target for drug development and a potential therapeutic strategy. Current research focuses on developing small-molecule compounds that specifically modulate the assembly of this complex, along with exploring the potential of restoring its function through gene therapy approaches. These studies not only enhance our understanding of the molecular mechanisms underlying autophagy but also offer new therapeutic targets for treating tumors, neurodegenerative disorders, and infectious diseases. A crucial mechanistic insight is that Beclin1 dynamically oscillates between inactive homodimers and active heterodimers formed with Vps34. Although structural studies have successfully mapped the key regulatory interfaces, it remains unknown whether botanical compounds can selectively disrupt the inhibitory Beclin1–Bcl-2 interaction without simultaneously affecting the functionally essential Beclin1–Vps34 association. Moreover, accumulated evidence indicates that autophagy deficiency induces inflammation via the TLR4/NLRP3 axis, but the precise mechanisms remain unclear.

#### 4.2.4 PPARα signaling pathway

The PPARα signaling pathway is a central regulator of cellular metabolism, playing a crucial role in lipid metabolism, energy homeostasis, and autophagy regulation (Lee et al., 2014). As a member of the nuclear receptor superfamily, PPARα can be activated by various ligands, including FAs, their derivatives, and fibrate drugs. It forms a heterodimer with the retinoid X receptor and binds to peroxisome proliferator response elements in the promoter regions of target genes, thereby regulating the transcriptional expression of downstream genes (Lazennec et al., 2000; Preidis et al., 2017). PPARα plays a multifaceted regulatory role in autophagy. Directly, it activates the transcription of several ATGs, including key molecules essential for autophagosome formation, such as LC3 and ATG7. Indirectly, PPARα influences the autophagic process by modulating lipid metabolism. It upregulates genes involved in fatty acid β-oxidation, such as CPT1A and ACOX1, thereby reducing intracellular lipid accumulation and mitigating lipotoxicity-induced inhibition of autophagic flux. Additionally, PPARα promotes the selective autophagic clearance of LDs by inducing the expression of genes associated with lipophagy (Shao et al., 2025). Notably, there is a close crosstalk between PPARα and energy-sensing pathways. Under fasting conditions, PPARα and AMPK form a positive feedback regulatory loop, in which AMPK phosphorylates and enhances PPARα activity, while PPARα upregulates AMPK expression. Together, they promote autophagy activation to maintain energy homeostasis (Dong et al., 2024). Furthermore, PPARα can alleviate mTORC1’s inhibitory effect on autophagy by suppressing its signaling activity, thereby establishing a coordinated regulatory network involving multiple pathways (Laplante and Sabatini, 2013). Dysregulation of this pathway is closely associated with the onset and progression of various metabolic diseases. In MAFLD, impaired PPARα function results in a reduced capacity for FA oxidation and autophagic dysfunction, exacerbating hepatic lipid accumulation and inflammatory responses (Tong et al., 2019). In cardiovascular diseases, reduced PPARα activity impairs the clearance of damaged mitochondria and oxidized lipids via the autophagy pathway, thereby promoting the progression of atherosclerosis (Cui et al., 2025). In neurodegenerative diseases, PPARα exerts a neuroprotective effect by regulating lipid metabolism in the brain and promoting autophagy (Ghannam et al., 2025).

PPARα plays a central role in regulating metabolism and autophagy, making it a crucial target for drug development in disease treatment. Fibrate drugs, which act as PPARα agonists, have demonstrated therapeutic efficacy in clinical applications by improving metabolic disorders through the modulation of autophagy (Yoo et al., 2021). The aim of the studies on novel selective PPARα modulators is to achieve more precise regulation of autophagy, offering new strategies for treating metabolic diseases. Therefore, an in-depth investigation of the specific mechanisms by which PPARα mediates autophagy in various tissues will facilitate the development of targeted and precise therapies for this pathway. An important functional consideration is that PPARα-mediated lipid clearance depends on the presence of functional lysosomal acid lipase to generate the free FAs required for β-oxidation. Therefore, if lysosomal acidification is impaired, which is a common pathological feature in MAFLD, PPARα agonism alone may prove ineffective. Furthermore, mitophagy must be precisely regulated as excessive clearance of mitochondria could paradoxically compromise ATP production in steatohepatitis, where energy metabolism is already under stress.

#### 4.2.5 HSC70/LAMP2A signaling pathway

The HSC70/LAMP2A CMA pathway is a crucial intracellular mechanism for the selective degradation of proteins, playing a vital role in maintaining protein homeostasis and cellular function. This pathway ensures precise protein quality control by specifically recognizing, transporting, and degrading substrate proteins that contain KFERQ-like motifs (Ikami et al., 2022). HSC70, the core molecular chaperone of this pathway, is responsible for recognizing specific sequences in substrate proteins and mediating their unfolding. In an ATP-dependent process, HSC70 forms a stable complex with the substrate protein and directs it to the lysosomal membrane. LAMP2A, a specific receptor on the lysosomal membrane, binds the HSC70–substrate complex through its cytoplasmic domain, assembling into a multimeric translocation channel. Through the cooperative action of molecular chaperones, the unfolded substrate protein passes through this channel into the lysosomal lumen, where it is subsequently degraded by hydrolases (Yang et al., 2019). The activity of this pathway is subject to precise, multi-level regulation. At the transcriptional level, transcription factors such as TFEB upregulate the expression of LAMP2A. At the post-translational level, the stability of LAMP2A is regulated by its distribution density on the lysosomal membrane, with higher density promoting the formation of stable multimeric transport complexes. Furthermore, intracellular nutritional status and stress signals dynamically modulate the pathway’s activity by regulating the degradation and recycling of LAMP2A. Notably, this signaling pathway has a complementary relationship with other forms of autophagy. When CMA is impaired, alternative pathways such as macroautophagy can be activated compensatorily (Signorelli et al., 2021; Ikami et al., 2022). Furthermore, dysregulation of this signaling pathway is strongly associated with multiple diseases. In neurodegenerative disorders, reduced expression of LAMP2A impairs the clearance of toxic proteins, such as α-synuclein, thereby accelerating disease progression (Alvarez-Erviti et al., 2013). In metabolic diseases, abnormal activity of this pathway disrupts the turnover of key proteins involved in the insulin signaling pathway, thereby exacerbating IR (Alicka et al., 2019). The gradual decline of this pathway during aging is strongly associated with the onset and progression of various age-related proteinopathies (Moreno-Blas et al., 2018). In tumor development, this pathway exerts dual effects by regulating the stability of both oncogenes and tumor suppressor genes (Dong R.-F. et al., 2025).

Given the central role of this pathway in maintaining protein homeostasis, regulation of the HSC70/LAMP2A signaling pathway has emerged as a novel strategy for disease treatment. Current research focuses on developing small-molecule compounds that enhance pathway activity, along with exploring the feasibility of restoring its function through gene therapy approaches. These studies not only deepen our understanding of selective autophagy mechanisms but also provide new therapeutic targets for related diseases. A key point to emphasize is that CMA activity critically depends on the multimerization of LAMP2A at the lysosomal membrane, a process influenced by the composition of lipid microdomains. However, it remains unclear whether CMA dysfunction in MAFLD primarily results from reduced transcription, potentially via impaired TFE3 signaling, or from defective multimerization at the membrane level. Additionally, the potential crosstalk between CMA and lipophagy, specifically whether CMA directly targets lipid droplet proteins for degradation, remains an unexplored area. Finally, human data on the gut–liver axis in this context are scarce, with most current knowledge derived from murine models.

#### 4.2.6 PINK1/Parkin signaling pathway

The PINK1/Parkin signaling pathway is a crucial regulatory mechanism of selective autophagy, playing a central role in the clearance of damaged mitochondria and the maintenance of cellular homeostasis (Narendra and Youle, 2024). The precise regulation of this pathway is essential for cellular survival, and its dysfunction is strongly associated with the onset and progression of various diseases. Under normal physiological conditions, PINK1 is imported into the inner mitochondrial membrane in a membrane potential-dependent manner and is rapidly degraded. When mitochondrial damage causes a loss of membrane potential, PINK1 stabilizes and accumulates on the outer mitochondrial membrane, where it becomes activated through autophosphorylation. Activated PINK1 phosphorylates ubiquitin molecules and the Parkin protein, inducing a conformational change in Parkin and recruiting it to the surface of the damaged mitochondria. Subsequently, Parkin catalyzes the ubiquitination of outer mitochondrial membrane proteins, with the resulting ubiquitin chains serving as recognition signals for autophagy receptor proteins such as OPTN and NDP52. This process, in turn, recruits autophagy-related proteins such as LC3, initiating the selective autophagic clearance of damaged mitochondria (Thayer et al., 2025). Notably, PINK1 can also directly phosphorylate ubiquitin chains through a Parkin-independent pathway to recruit autophagy receptors, thereby facilitating mitochondrial clearance (Nguyen et al., 2023). In addition to mitophagy, this signaling pathway also regulates several important physiological processes, including mitochondrial biogenesis, inflammatory immune responses, and cellular metabolic adaptation (He et al., 2021; Tian et al., 2025). Meanwhile, abnormalities in this signaling pathway are closely associated with various diseases. In Parkinson’s disease, mutations in the PINK1 or Parkin genes impair mitophagy, leading to the accumulation of damaged mitochondria and the degeneration of dopaminergic neurons (Jiang et al., 2024). During tumorigenesis, dysregulation of this signaling pathway can compromise genomic stability and alter the metabolic adaptability of tumor cells (Luo et al., 2024). Furthermore, in metabolic and cardiovascular diseases, disruption of the PINK1/Parkin signaling pathway is closely associated with tissue dysfunction (Cui et al., 2018; Zhao X. et al., 2024).

**FIGURE 4:**
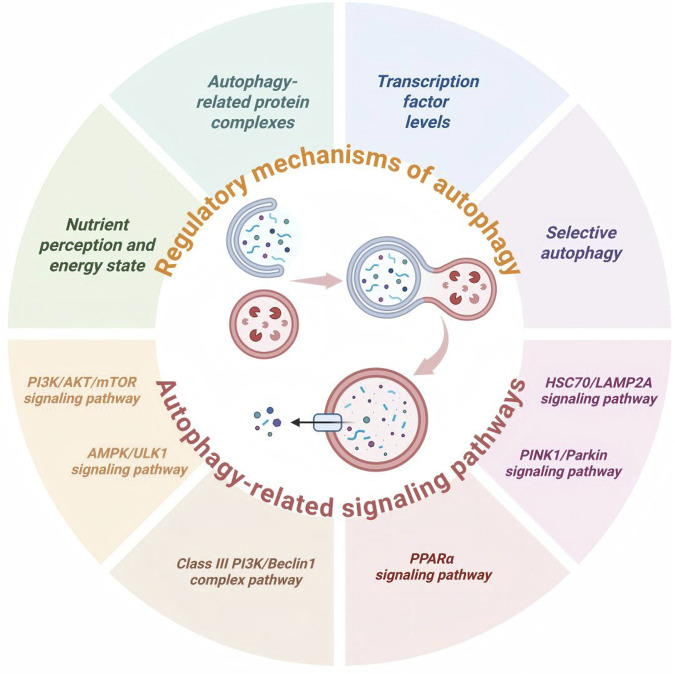
Regulatory mechanism and related signaling pathway of autophagy, including nutrient perception and energy state, autophagy-related protein complexes, transcription factor levels, selective autophagy, the PI3K/AKT/mTOR signaling pathway, the AMPK/ULK1 signaling pathway, the Class III PI3K/Beclin1 complex pathway, the PPARα signaling pathway, the PINK1/Parkin signaling pathway, and the HSC70/LAMP2A signaling pathway.

In conclusion, the PINK1/Parkin signaling pathway plays a crucial role in maintaining cellular homeostasis. Intervention strategies targeting this pathway have emerged as promising therapeutic approaches for various diseases. Current research priorities include developing small-molecule agonists to enhance pathway activity and exploring gene therapy techniques to restore function. These studies not only deepen our understanding of autophagy regulatory networks but also offer novel insights for developing innovative treatments for a wide range of human diseases. A critical consideration is that the PINK1/Parkin pathway exhibits cell-type-specific kinetics. For example, in hepatocytes, receptor-mediated mitophagy involving BNIP3 and NIX may predominate under lipotoxic stress conditions. Additionally, Parkin recruitment to damaged mitochondria requires prior phosphorylation of ubiquitin, and the availability of ubiquitin itself may become limiting in lipid-overloaded hepatocytes. Therefore, any potential mitophagy enhancers must be rigorously evaluated for their ability to overcome these specific bottlenecks, rather than simply increasing Parkin expression. The multi-target nature of many phytochemicals further complicates both mechanistic analysis and dose optimization (Figure 4).

## 5 Correlation between MAFLD and autophagy

### 5.1 Autophagy in the pathological progression of MAFLD

Autophagy is a critical, context-dependent determinant of MAFLD pathogenesis, with impaired autophagic flux contributing to disease progression at multiple stages. MAFLD is characterized by hepatocellular lipid overload, IR, inflammation, and progressive fibrosis (Mejía-Guzmán et al., 2025). By mediating lipophagy, mitophagy, and ER-phagy, autophagy facilitates the clearance of LDs, the removal of damaged organelles, and the maintenance of metabolic homeostasis. Failure of these selective autophagic processes accelerates MAFLD progression (Chen and Lin, 2022). Autophagy can be disrupted at various stages in MAFLD. Initiation is commonly inhibited by hyperactive mTOR signaling or impaired AMPK activity. Autophagosome biogenesis can be compromised by altered expression of ATGs and by physical or regulatory constraints imposed by LD-coating proteins such as PLIN2. Downstream, autophagosome–lysosome fusion and cargo degradation are frequently impaired due to defective lysosomal acidification and hydrolase function (Chi et al., 2024). Lipid overload further exacerbates these defects: short-chain fatty acids (SCFAs) can impair autophagic flux, inhibit autophagosome–lysosome fusion, and promote accumulation of SQSTM1/p62, creating a lipotoxic feed-forward loop that worsens hepatocyte injury (Cho et al., 2018). IR, as an upstream driver of MAFLD, can suppress autophagy via transcription factor networks (e.g., FOXO and TFEB), reducing lipid clearance and promoting LD accumulation (Zhang L. et al., 2025; Sha et al., 2024). Environmental and genetic factors converge on the same axis: high-fat diet (HFD) suppresses hepatocyte autophagy, aggravating steatosis and mitochondrial dysfunction, while elevated free fatty acids (FFAs) disrupt the autophagy-lysosome pathway through ER-stress signaling (Naito et al., 2019). Genetic evidence further supports causality as reduced ATG5/ATG7 function correlates with disease severity. The PNPLA3 I148M variant impairs LD remodeling and autophagic degradation, whereas the TM6SF2 E167K variant may disturb the balance between VLDL secretion and autophagic flux (Li et al., 2023; Naito et al., 2019).

Autophagy plays a dual role in MAFLD. Appropriate activation promotes the clearance of LDs, improves insulin sensitivity, and reduces inflammation, whereas severe inhibition—or potentially maladaptive activation—can disrupt metabolic homeostasis. Therapeutically, targeting the autophagy network—such as enhancing lipophagy and restoring lysosomal function through nutritional interventions, small molecules, or gene-based approaches—represents a rational strategy. Future research should elucidate the stage-specific dynamics of selective autophagy pathways in MAFLD to enable more precise, mechanism-driven interventions. It is crucial to recognize that autophagic defects in MAFLD are inherently cell-type-specific. Although hepatocyte autophagy primarily regulates lipid metabolism, autophagy in Kupffer cells and hepatic stellate cells plays distinct roles in modulating inflammation and fibrosis. This complexity underscores the need for studies at single-cell resolution. Furthermore, the selective clearance of different cargoes, such as lipids versus mitochondria, may be differentially impaired in the diseased liver, necessitating the development of cargo-specific flux measurements. Additionally, the presence of a lipophagy–SREBP-1c feedback loop requires stage-specific targeting strategies to avoid the unintended consequence of compensatory lipogenesis.

### 5.2 Pathological connection between autophagy and MAFLD

**FIGURE 5:**
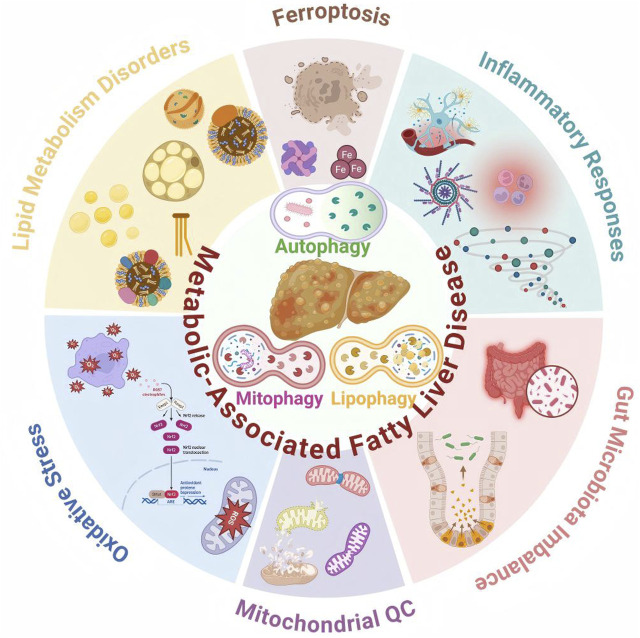
Potential pathological mechanisms linking autophagy to MAFLD include inflammatory responses, lipid metabolism disorders, mitochondrial quality control system dysfunction, oxidative stress, gut microbiota imbalance, and ferroptosis. MAFLD, metabolic-associated fatty liver disease.

MAFLD is one of the most prevalent chronic liver diseases worldwide and is particularly common in East Asia, where MetS is highly prevalent. Its onset and progression involve complex molecular pathways and dysregulation of cellular death mechanisms. Autophagy, a conserved lysosome-dependent degradation and recycling process, plays a key role in the progression of MAFLD toward steatohepatitis and fibrosis. Potential mechanistic links include inflammatory responses, lipid metabolism disorders, mitochondrial quality control dysfunction, oxidative stress, gut microbiota imbalance, and ferroptosis (Figure 5). Defining autophagy-regulatory networks in MAFLD, along with their crosstalk with metabolic reprogramming and the inflammatory microenvironment, will help refine disease models and inform translational strategies to therapeutically target autophagy.

#### 5.2.1 Autophagy and inflammatory responses

Autophagy and inflammation form a bidirectional, self-reinforcing network in MAFLD that influences disease progression. Autophagy is generally anti-inflammatory by clearing lipid droplets, damaged mitochondria, and the ER, thereby limiting lipotoxicity and oxidative stress. It can also suppress inflammasome activity. For example, autophagic degradation of NLRP3 inflammasome metabolites reduces the maturation and release of IL-1β and IL-18. Conversely, inflammatory signals can modulate autophagic flux: TNF-α and IL-6 may inhibit autophagy via NF-κB or JNK signaling pathways, whereas anti-inflammatory mediators can enhance autophagy to support metabolic homeostasis (Matsuzawa-Ishimoto et al., 2018).

In MAFLD, impaired autophagy and hepatic inflammation often establish a feed-forward loop. Defective autophagy reduces lipid clearance and promotes the accumulation of lipotoxic species (e.g., ceramides and lysophosphatidylcholine), which activate innate immune pathways, including TLRs and the NLRP3 inflammasome, in Kupffer cells and hepatic stellate cells (HSCs). This activation drives persistent inflammation and cytokine production (An et al., 2023). Conversely, chronic inflammation further impairs lipophagy and mitophagy by limiting TFEB nuclear translocation, disrupting lysosomal function, and hindering autophagosome maturation, thereby amplifying a metabolic–inflammatory vicious cycle (Zhang et al., 2019). Genetic risk variants can converge on this axis; for example, the PNPLA3 I148M variant is associated with impaired autophagic flux, alongside increased inflammatory signaling (Luukkonen et al., 2023), while the TM6SF2 E167K variant disrupts VLDL secretion and autophagic balance, exacerbating hepatic inflammation (Borén et al., 2020).

These interactions highlight the autophagy–inflammation interface as a promising therapeutic target in MAFLD. Enhancing autophagy (e.g., through mTORC1 inhibitors, AMPK agonists, or TFEB activators) may indirectly reduce hepatic inflammation by improving lipid metabolism. Conversely, anti-inflammatory strategies (e.g., IL-1β blockade or FXR agonism) may help restore autophagic function, thereby collectively slowing disease progression. Furthermore, elucidating cell-type-specific signaling pathways among hepatocytes, Kupffer cells, and HSCs should facilitate mechanism-driven approaches that simultaneously modulate autophagy and inflammation. It is important to note that the anti-inflammatory effects of autophagy are highly cell-type-dependent. In hepatocytes, autophagy limits lipid-induced inflammation, whereas in Kupffer cells, it directly suppresses inflammasome activation. This cell-type specificity suggests that non-selective enhancement of autophagy could produce opposing outcomes. Additionally, chronic inflammation can induce autophagy resistance via sustained NF-κB activation, potentially requiring anti-inflammatory pretreatment before autophagy-modulating therapies become effective. Finally, stage-specific immune responses must be carefully considered to avoid unintended immunosuppression.

#### 5.2.2 Autophagy and lipid metabolism disorders

Autophagy and lipid dysmetabolism engage in a bidirectional regulatory loop that is central to the pathogenesis of MAFLD. Lipotoxic species—such as FFAs, ceramides, and oxidized lipids—remodel hepatocyte autophagy. SCFAs can suppress autophagy initiation by inhibiting AMPK and activating mTOR signaling, whereas polyunsaturated fatty acids (PUFAs) may enhance autophagy via the SIRT1/FOXO1 signaling pathway (Marcondes-de-Castro et al., 2023). Additionally, cholesterol overload destabilizes lysosomal membranes and can directly impair the completion of autophagic flux (Appelqvist et al., 2011). Conversely, autophagy is a key determinant of lipid homeostasis. Lipophagy mediates LD turnover and TAG mobilization, and intact hepatocyte ATG networks—such as ATG5, ATG7, and TFEB—support lipid balance, insulin sensitivity, and protection from lipotoxicity (Xu and Fan, 2022; Zhao et al., 2023). CMA can further contribute to LD regulation and lipid handling (Mastoridou et al., 2023).

In MAFLD, disruption of the autophagy–lipid axis drives disease progression. Hepatocellular lipid overload induces lipotoxic stress, which promotes IR and mitochondrial dysfunction. Concurrent impairment of autophagy further amplifies LD accumulation and metabolic injury. Lipotoxic oxidative and ER stress can additionally inhibit autophagic flux, establishing a feed-forward vicious cycle (Jana et al., 2019; Singh et al., 2009). Consistently, MAFLD models often exhibit reduced LC3-II levels alongside SQSTM1/p62 accumulation, indicative of impaired autophagic flux. Restoring autophagy—through caloric restriction or pharmacological interventions—can improve hepatic steatosis and IR in preclinical models (Deng et al., 2025; Bhatnagar et al., 2025; Zhang et al., 2024).

Therapeutically, targeting the autophagy–lipid interface holds great promise and encompasses lifestyle interventions (such as diet and exercise), along with pharmacological agents that modulate lipid metabolism and autophagy (e.g., AMPK agonists and FXR ligands). Future research should clarify how distinct lipid species regulate autophagy and characterize stage-specific alterations as MAFLD progresses to steatohepatitis and fibrosis, thereby enabling precision strategies based on autophagic regulation. It is important to recognize that different lipid species exert opposing effects on autophagy: saturated fats tend to inhibit autophagic flux, whereas unsaturated fats may help preserve it. Consequently, the overall impact of lipid overload on autophagy critically depends on the dietary fatty acid composition. This nuance suggests that the concept of “lipotoxicity” should be refined to distinguish between autophagy-suppressive and autophagy-permissive lipids. Moreover, considering the lipophagy–SREBP-1c axis, stage-specific targeting is necessary to prevent the activation of compensatory lipogenic pathways.

#### 5.2.3 Autophagy and mitochondrial quality control system dysfunction

Autophagy and the mitochondrial quality control system are reciprocally linked and together influence the pathogenesis of MAFLD. The mitochondrial quality control system maintains mitochondrial function through mitophagy, biogenesis, and dynamics, while autophagy—particularly mitophagy—is the primary mechanism for removing dysfunctional mitochondria (Wang and Wang, 2019).

During the progression of MAFLD, this regulatory circuitry is often disrupted. IR and an excessive influx of FFAs drive an overload of fatty acid β-oxidation, increasing ROS and causing a loss of mitochondrial membrane potential (ΔΨm). This loss should trigger PINK1/PRKN (Parkin)-dependent mitophagy to clear damaged mitochondria. However, if this pathway is compromised, dysfunctional mitochondria accumulate, amplifying oxidative stress and lipid peroxidation, thereby establishing a feed-forward loop of hepatocyte injury (Jiang et al., 2021). Lipotoxicity can further inhibit autophagic initiation and completion by suppressing AMPK signaling and activating mTOR pathways (Zhang S. et al., 2018). Conversely, impaired autophagic flux promotes accumulation of FA-ox intermediates, and reduced expression of mitophagy-related genes limits hepatocyte metabolic plasticity (Liu S. et al., 2025). The lipid peroxidation product 4-hydroxynonenal (4-HNE) exacerbates this cycle by damaging mitochondrial DNA and proteins and by interfering with mitophagy signaling and cargo recognition (Xiao et al., 2017). Overnutrition and HFD can additionally uncouple mitochondria and alter membrane permeability, destabilizing the coordination between the mitochondrial quality control system and autophagy (Souza et al., 2022).

These insights have driven the development of therapeutic strategies aimed at restoring mitochondrial quality control and mitophagy in MAFLD. Such strategies include the use of AMPK activators (e.g., metformin), mitochondria-targeted antioxidants to mitigate oxidative damage, and nutritional interventions that reactivate autophagic flux. Defining the stage-specific crosstalk between mitochondrial quality control and autophagy throughout MAFLD progression is essential to guide the development of metabolism-protective therapies designed to halt disease advancement. A critical observation is that mitophagy exhibits distinct threshold effects: mild mitochondrial stress typically triggers adaptive mitophagy to restore homeostasis, whereas severe stress can overwhelm autophagic capacity, ultimately leading to cell death. This threshold is influenced by factors such as receptor availability and lysosomal function. However, a major unresolved question remains: whether impaired mitophagy in a given context reflects failure at the cargo recognition step—mediated by the PINK1/Parkin pathway—or at the distal fusion step. These two scenarios have fundamentally different therapeutic implications, yet they remain undifferentiated in current research. Another concern is that excessive mitophagy, if not properly regulated, may compromise ATP production during the energy-demanding state of steatohepatitis.

#### 5.2.4 Autophagy and oxidative stress

Autophagy and oxidative stress are reciprocally regulated in MAFLD, and their crosstalk drives hepatocellular metabolic injury and inflammatory activation. Oxidative stress results from excessive ROS production or insufficient antioxidant capacity, leading to lipid peroxidation, protein oxidation, and DNA damage. Autophagy functions as a quality control mechanism that mitigates these insults by removing damaged mitochondria and oxidized protein aggregates (Gao, 2019). In MAFLD, this balance is often disrupted. Increased fatty acid β-oxidation flux, lipotoxicity, and mitochondrial dysfunction elevate ROS levels, which can oxidatively modify components of the autophagic machinery (e.g., ATG4 and Beclin-1), impairing autophagic initiation and flux completion. Conversely, defective autophagy promotes the accumulation of dysfunctional mitochondria and lipid peroxidation products (e.g., MDA and 4-HNE), further amplifying ROS generation and establishing a feed-forward loop (Filomeni et al., 2015).

This crosstalk is embedded within metabolic signaling pathways. ROS can activate AMPK and inhibit mTOR, thereby inducing autophagy as an adaptive response. However, sustained excess ROS may lead to maladaptive hyperactivation of autophagy and, in some contexts, autophagy-associated cell death (Li L. et al., 2013). Autophagy, in turn, mitigates oxidative stress through the selective clearance of damaged mitochondria (mitophagy) and LDs (lipophagy) (Long and McWilliams, 2023). Metabolic dysfunction associated with MAFLD further converges on this axis: IR can coordinately alter antioxidant programs and autophagy via FOXO signaling, while chronic SREBP1-driven lipogenesis can suppress autophagic capacity (Nguyen et al., 2021).

Therapeutically, the interface between autophagy and oxidative stress represents a promising target in MAFLD. Antioxidants such as vitamin E and N-acetylcysteine can reduce oxidative damage and have been reported to support autophagic competence (Aslan et al., 2025; Chen Q. et al., 2025). Meanwhile, autophagy inducers such as metformin may indirectly decrease ROS by restoring mitochondrial quality control and clearing oxidized proteins (Cai L. et al., 2025). Omega-3 fatty acids, specifically DHA and EPA, have demonstrated antioxidative and anti-apoptotic effects in renal ischemia/reperfusion injury, and their potential to confer cross-organ protection mediated by autophagy has been proposed (Ajami et al., 2013). Defining stage- and cell-type-specific mechanisms—for example, in hepatocytes and Kupffer cells—should inform precision strategies that co-modulate redox stress and autophagic flux. It is important to recognize that ROS play a biphasic role in autophagy regulation. At low levels, ROS can activate autophagy through pathways involving AMPK and the oxidation of ATG4. In contrast, high ROS levels can oxidatively damage autophagic proteins, thereby impairing autophagic flux. This dual role suggests that moderate antioxidant supplementation may help preserve autophagic function, whereas excessive antioxidant use could blunt these essential adaptive responses. The source of ROS—whether mitochondrial or endoplasmic reticulum-derived—also determines which specific autophagic pathways are affected. Furthermore, oxidized lipids can directly modify lysosomal membrane proteins, such as LAMP2A, leading to impaired CMA and autophagosome–lysosome fusion.

#### 5.2.5 Autophagy and gut microbiota imbalance

Autophagy and the gut microbiota reciprocally regulate one another within the gut–liver axis, and this crosstalk is critically implicated in the pathogenesis of MAFLD. Microbiota-derived metabolites, including short-chain fatty acids, secondary bile acids, and trimethylamine, can modulate hepatic autophagy (Lapaquette et al., 2021). For example, butyrate activates AMPK and enhances autophagy, thereby supporting hepatocyte lipid metabolism (Miao et al., 2025). In contrast, lipopolysaccharides (LPSs) derived from Gram-negative bacteria activate innate immune pathways such as TLR4/NF-κB and inflammasome signaling in Kupffer cells, amplifying cytokine production. These inflammatory signals are associated with impaired autophagic function (Kong et al., 2017; Fan et al., 2021).

Autophagy plays a crucial role in maintaining intestinal homeostasis. Proper functioning of ATGs in intestinal epithelial cells supports barrier integrity and helps regulate bile acid metabolism and lipid absorption (Apte, 2021). In MAFLD, dysbiosis and barrier dysfunction increase intestinal permeability, enhancing the portal delivery of microbial products to the liver and sustaining hepatic immune activation. Additionally, altered bile acid pools can modulate hepatocyte autophagy through the FXR and TGR5 pathways (Khambu et al., 2019). Impaired autophagy further exacerbates this gut–liver axis disturbance by reducing lipid droplet clearance and promoting steatosis, thereby reinforcing a feed-forward cycle of metabolic stress and inflammation (Cho et al., 2023; Liu et al., 2021). In MAFLD models, shifts in the microbiota are often accompanied by reduced LC3-PE (LC3-II) levels and accumulation of SQSTM1/p62, consistent with impaired autophagic flux. Conversely, probiotics or dietary fibers have been reported to enhance autophagy and improve steatosis (Seif El-Din et al., 2021). Specific microbial metabolites, including ursodeoxycholic acid and indole derivatives, are also associated with hepatoprotection, partly through autophagy modulation (Liu X. et al., 2025; Shaheen et al., 2025).

These data support therapeutic strategies targeting the microbiota–autophagy interface in MAFLD, including the use of prebiotics, probiotics, and dietary interventions to reprogram microbial ecology, along with small molecules designed to restore autophagic competence. Future research should elucidate strain- and metabolite-specific mechanisms and define cell-type-specific responses along the gut–liver axis to enable precision interventions for MAFLD. A significant limitation in the field is the lack of causal evidence demonstrating that specific microbiota changes directly drive alterations in hepatic autophagy in MAFLD; most available data are merely correlational. Although microbial metabolites such as butyrate have been shown to modulate autophagy *in vitro*, their actual concentrations in the portal vein and *in vivo* relevance in human patients require rigorous pharmacokinetic validation. Interpretation of these interactions is further complicated by the fact that the gut microbiota itself is regulated by intestinal autophagy, creating a complex bidirectional loop. Finally, well-controlled human studies investigating this axis remain scarce, with most insights derived from animal models.

#### 5.2.6 Autophagy and ferroptosis

Autophagy and ferroptosis are distinct yet interconnected processes that collectively contribute to hepatocyte injury in MAFLD. Autophagy, including lipophagy, maintains metabolic homeostasis by clearing LDs and damaged organelles. In MAFLD, impaired autophagic flux reduces lipid clearance and exacerbates lipotoxicity. Ferroptosis is an iron-dependent form of cell death driven by phospholipid peroxidation, characterized by the loss of GPX4 activity, collapse of antioxidant defenses, and accumulation of lipid peroxides (Dixon and Olzmann, 2024). In MAFLD, hepatic iron overload combined with lipid peroxidation creates a permissive environment for ferroptosis, thereby promoting hepatocyte injury and inflammatory activation (Shao and Chung, 2024).

Mechanistically, autophagy modulates ferroptosis sensitivity through ferritinophagy, the selective degradation of ferritin that increases labile iron and enhances lipid peroxidation. Conversely, excessive or maladaptive autophagy may promote ferroptosis by impairing antioxidant capacity or inducing mitochondrial dysfunction. Ferroptotic lipid peroxides and oxidative stress products can, in turn, compromise autophagic function, creating a feed-forward loop of metabolic injury (Zhao S. et al., 2024). In MAFLD, IR and excess FFAs simultaneously suppress autophagy and promote iron accumulation, while lipotoxic oxidative stress is both a consequence of autophagic failure and a driver of ferroptosis (Yoon et al., 2021).

These insights highlight the autophagy–ferroptosis interface as a promising therapeutic target. Activation of autophagy can be beneficial; for example, metformin enhances autophagy via AMPK signaling and promotes TFEB nuclear translocation, thereby improving HFD-induced steatosis and IR in mice (Park et al., 2023). Concurrently, ferroptosis-targeted interventions demonstrate hepatoprotective potential. For instance, rociletinib (ROC), which targets ACSL4, has been reported to attenuate ferroptosis-driven acute liver injury (Linghu et al., 2025), and dietary oleic acid can reduce peroxidation-prone PUFA phospholipids in membranes, thereby limiting ferroptosis (Xiao et al., 2023). Accordingly, co-targeting autophagy and ferroptosis—by restoring intracellular clearance while blocking lipid peroxidation injury—has shown synergistic benefits in experimental models and warrants further evaluation in MAFLD. Defining the stage-specific dynamics of this axis during progression from steatosis to steatohepatitis and fibrosis should inform multi-target strategies tailored to MAFLD progression. A fundamental paradox exists at the autophagy–ferroptosis interface. Basal ferritinophagy, a selective autophagic process, provides the labile iron pool required for ferroptosis to occur. However, stress-induced autophagy can either protect against or promote ferroptosis, depending on the specific cargo being degraded. This creates a therapeutic paradox: non-selective enhancement of autophagy might inadvertently increase ferroptosis risk through excessive ferritin degradation. Conversely, global autophagy inhibition would block ferritinophagy and impair the protective functions of lipophagy and mitophagy. This intricate relationship suggests that selectively modulating ferritinophagy—for instance, by targeting NCOA4—may represent a more refined therapeutic approach. Although autophagy deficiency is known to promote ferroptosis, it is also important to note that inhibiting ferroptosis could potentially impair lipid clearance, highlighting the need for a balanced strategy.

## 6 Targeting autophagic flux to delay the progression of MAFLD

**FIGURE 6:**
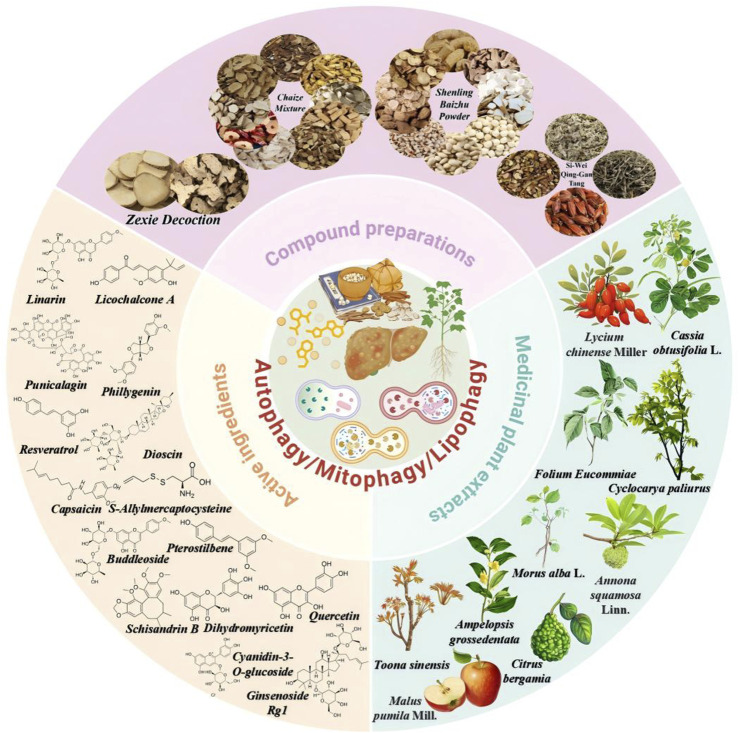
TCM compound preparations, medicinal plant extracts, and their bioactive compounds with autophagy potential for the treatment of MAFLD.

Natural products derived from medicinal plants are increasingly recognized as multi-constituent, multi-target, and multi-pathway agents—a pharmacological profile that aligns with the multifactorial biology of MAFLD (Lu et al., 2025). Experimental studies indicate that herbal extracts and purified constituent can exert antioxidant, anti-inflammatory, lipid-lowering, and insulin-sensitizing effects, often demonstrating favorable safety profiles in both preclinical and clinical settings (Ashkar et al., 2020; Chan and Tomlinson, 2000; Ge et al., 2021; Verma et al., 2021; Zheng et al., 2025). Notably, multiple bioactive classes common in Chinese herbal medicine—including flavonoids, polyphenols, saponins, and alkaloids—have been reported to modulate autophagic flux during MAFLD progression. Mechanistically, these effects converge on autophagy-related nodes, including the PI3K/AKT/mTOR, AMPK/TFEB, PINK1/Parkin, ULK1/Beclin-1/VPS34, p62/Nrf2, IκBα/NF-κB, and miR-375-3p/ATG2B/PTEN/AKT pathways (Tables 1–3). Collectively, these findings provide a strong rationale for leveraging natural products to modulate autophagy in MAFLD and support continued investigation of pathway-specific mechanisms to guide the development of autophagy-targeted modulators derived from natural sources (Figure 6).

**TABLE 1:** Multi-herb TCM formulas that modulate autophagy-related pathways in MAFLD models.

Chinese herbal compound preparation	Dosage/Route/Time	Autophagy modulator/Positive control	Related target	Pharmacological mechanism	*In vitro*/*In vivo*	Model	Reference
Zexie Decoction	4.31 g/kg/day, intragastric administration for 12 weeks	Not mentioned	SOD, MDA, α-SMA, and LC3B	Modulating oxidative stress and autophagy	*In vivo*	MCD-induced male C57BL/6 mouse model	Xu et al. (2019)
Chaize Mixture	0.57–1.14 mg/mL, medicated culture medium for 24 h; 5.915–11.83 g/kg/day, intragastric administration for 4 weeks	Silybin	GLIPR2, LC3B, p62, Beclin1, NLRP3, Caspase-1, IL-1β, α-SMA, CPT1A, SREBP1, Fas, TNF-α, IL-6, Timp1, Timp2, TGF-β, Acta2, and Adamstl2	Promoting autophagy and inhibiting NLRP3-mediated inflammation, which was achieved by regulating GLIPR2	*In vitro* and *in vivo*	OA/LPS-induced AML-12 cell injury model; MCD-induced male C57BL/6J mouse model	Zhang et al. (2025b)
Shenling Baizhu Powder	0.5 mM, medicated culture medium for 24 h; 30 mg/kg/day, intragastric administration for 6 weeks	Rapamycin	SIRT1, LC3B, Beclin1, ATG5, p62, CAT, AKT, p-AKT, HO-1, HIF-1α, eNOS, and NQO1	Inducing autophagy activation and ultimately alleviating oxidative stress, endoplasmic reticulum stress, and mitochondrial dysfunction	*In vitro* and *in vivo*	OA-induced HepG2 cell injury model; HFD-induced male Wistar rat model	Pan et al. (2024)
Modified Suanmei-Tang	0.55, 1.1, and 2.2 g/kg/day, intragastric administration for 12 weeks	Metformin	TNF-α, IL-1β, Beclin1, LC3B, p62, PI3K, p-PI3K, AKT, p-AKT, mTOR, and p-mTOR	Regulating the PI3K/AKT/mTOR pathway to improve autophagy	*In vivo*	HFD-induced male C57BL/6J mouse model	Wang et al. (2024b)
Si-Wei-Qing-Gan-Tang	1–3 g/kg/day, intragastric administration for 4 weeks	Polyene phosphatidylcholine capsule	TNF-α, IL-1β, IL-6, TLR4, p-AKT, p-mTOR, p-NF-κB p65, p-p38 MAPK, p-MEK1/2, p-ERK1/2, p-mTOR, p62, p-ULK1, and LC3B	Downregulation of NF-κB and activation of autophagy	*In vivo*	MCD-induced male SD rat model	Xiao et al. (2020)
Ger-Gen-Chyn-Lian-Tang	50–150 mg/kg/day, intragastric administration for 28 days	Not mentioned	Tmem26, Tfam, Prdm16, SREBP-1c, Histone, CD36, HIF-1α, VEGF, MMP9, MCP1, SIRT1, PGC1α, UCP1, Parkin, PINK1, LC3B, p62, p-p62, MNF1, DRP1, and p-DRP1	Protecting lipotoxicity and ameliorating inflammation signaling through regulation of mitochondrial biogenesis and mitophagy	*In vivo*	C57BL/6J and BKS.Cg-m ^+/+^ Lepr db/JNarl (db/db) mouse models	Wang et al. (2022a)
Jiang Zhi Granule	100 μg/mL, medicated culture medium for 24 h; 994 mg/kg/day, intragastric administration for 3 weeks	Rapamycin	LC3B, p-PI3K, p-AKT, AKT, p-IRS-1, IRS-1, p-mTOR, mTOR, and p62	Activating autophagy through the mTOR signaling pathway	*In vitro* and *in vivo*	PA-induced primary hepatocyte injury model; MCD-induced C57B/6J male mouse model	Zheng et al. (2018)

TCM, traditional Chinese medicine; MAFLD, metabolic-associated fatty liver disease; MCD, methionine–choline-deficient, OA, oleic acid; LPS, lipopolysaccharide; HFD, high-fat diet; SD, Sprague–Dawley; PA, palmitoleic acid.

**TABLE 2:** Extracts from individual medicinal plants (single-herb extracts) reported to modulate autophagy in MAFLD models.

Single-herb extract	Medicinal plant	Dosage/Route/Time	Autophagy modulator/Positive control	Related target	Pharmacological mechanism	*In vitro*/*In vivo*	Model	Reference
*Lycium barbarum* polysaccharides	*Lycium chinense* Mill. [Solanaceae; fructus]	2 μM, 3 mM, medicated serum for 24 h; 1 mg/kg/day, intragastric administration for 4 weeks	Not mentioned	Resistin, p-IRS-1, p-GSK3α, p-SMAD2, p-SMAD4 NTR, IkBα, p-mTOR, Beclin1, ATG5, LC3B, p62, cleaved Caspase3, Cyto c, p-p38, p-JNK, and p-ERK1/2	Reduced insulin resistance, serum aminotransferases, inflammatory responses, apoptosis, and induced autophagy	*In vitro* and *in vivo*	PA-induced BRL-3A cell injury model; HFD-induced female SD rat model	Xiao et al. (2014)
*Cassiae Semen* extract	*Cassia obtusifolia* L. [Fabaceae; semen]	100, 200, and 400 mg/kg/day, intragastric administration for 4 weeks	Not mentioned	pre-SREBP1, SREBP1c, FASN, ACOX1, CPT1A, p-AMPK, p-mTOR, p-ULK, LC3B, ATG5, p62, and TFEB	Decreasing FASN-related fatty acid synthesis and activating AMPK-mediated autophagy	*In vivo*	HFSW-induced male C57BL/6J mouse model	Ding et al. (2023)
*Eucommia ulmoides* leaf extract	*Eucommia ulmoides* Oliv. [Eucommiaceae; folium]	25, 50, and 100 μg/mL, medicated culture medium for 24 h	Simvastatin, rapamycin, and chloroquine	PPARγ and LC3B	Lowering lipids by regulating PPARγ expression through inducing autophagy	*In vitro*	OA-induced HepG2 cell injury model	Gong et al. (2022)
*Cyclocarya paliurus* extract	*Cyclocarya paliurus* (Batalin) Iljinsk. [Juglandaceae; folium]	6.25, 12.5, 25, 50, and 100 μg/mL, medicated culture medium for 24 h	Rapamycin	LC3B, Beclin1, p-mTOR, mTOR, p-p70S6K, p70S6K, and p62	Inducing hepatic fat clearance through the autophagy–lysosome pathway, known as lipophagy	*In vitro*	OA- and PA-induced HepG2 cell injury model	Yang et al. (2020b)
Fermented mulberry leaves	*Morus alba* L. [Moraceae; folium]	50 mg/kg/day, intragastric administration for five times a week, 12 weeks	Not mentioned	PPAR-γ, C/EBPα, FAS, aP2, Klf2, iNOS, COX-2, p-JNK, JNK, p-ERK, ERK, p-p38, p38, IL-1β, IL-6, TNF-α, NF-κB, p-PI3K, PI3K, p-AKT, AKT, p-mTOR, mTOR, Beclin1, and LC3B	Regulation of the inflammatory response and autophagy pathway	*In vivo*	HFD-induced C57BL/6N male mouse model	Lee et al. (2020)
*Annona muricata* (Graviola) extract	*Annona squamosa* Linn. [Annonaceae; folium]	50 and 100 mg/kg/day, intragastric administration for 9 weeks	Not mentioned	FBG, HbA1c, HOMA-IR, 4-HNE, protein carbonyls, Nrf2, NQO1, p-AMPK, p-mTOR, LC3B, FAS, SREBP1c, C/EBPα, PPARγ, CPT1A, PPARα, p-AMPK, and PGC1α	Regulation of insulin signaling associated with energy metabolism and autophagy	*In vivo*	HFD with two-times STZ injection in C57BL/6 male mice	Son et al. (2021)
Vine tea total flavonoids	*Ampelopsis grossedentata* (Hand.-Mazz.) W.T.Wang [Vitaceae; folium]	5, 10, 20, 40, 80, and 160 μg/mL, medicated serum for 24 h; 125 and 250 mg/kg/day, intragastric administration for 6 weeks	3-Methyladenine	SREBP1, FASN, ACC, LC3B, p62, AMPK, p-AMPK, p-mTOR, and p-ULK1	Activation of AMPK/mTOR, leading to the stimulation of autophagy and a decrease in the buildup of intracellular lipids	*In vitro,* *in vivo*	OA-induced HepG2, L02 cell injury model; HFD-induced C57BL/6J mouse model	Wang et al. (2024a)
Bergamot polyphenol fraction	*Citrus bergamia* Risso [Rutaceae; fructus]	50 mg/kg/day, intragastric administration for 3 months	Not mentioned	LC3B, SQSTM1, ADRP, and Beclin1	Reducing serum triglyceride, blood glucose, and hepatic steatosis while inducing autophagy	*In vivo*	CAF-induced female Han Wistar rat model	Parafati et al. (2015)
Polyphenol-rich extracts	*Toona sinensis* (A. Juss.) M. Roem. [Meliaceae; cortex, fructus]	100, 200, and 500 μg/mL, medicated culture medium for 24 h	Chloroquine	FASN, p-AMPK, AMPK, p-ACC, ACC, SREBP-1c, PPARα, SCD1, LXR, and LC3B	Ameliorating free fatty acid-induced lipogenesis through AMPK and LC3 pathways	*In vitro*	FFA-induced HepG2 cell injury model	Chen et al. (2019)
Apple polyphenol extract	*Malus pumila* Mill. [Rosaceae; fructus]	10, 20, 30, 40, and 80 μg/mL, medicated culture medium for 24 h	Chloroquine and leupeptin	SREBP-1c, ACC, p-ACC, FASN, SCD1, PPARα, CPT1A, PGC-1α, SIRT1, LKB1, p-AMPK, p-ULK1, p-mTOR, ATG7, LC3B, p62, RAB7, Beclin1, TFEB, and LAMP1/2	Activating autophagy mediated by the SIRT1/AMPK signaling pathway	*In vitro*	PA/OA-induced HepG2 cell injury model	Li et al. (2021a)

MAFLD, metabolic-associated fatty liver disease; PA, palmitoleic acid; HFD, high-fat diet; SD, Sprague–Dawley; HFSW, high-fat and sugar-water; OA, oleic acid; STZ, streptozotocin; CAF, cafeteria diet (15% fat), FFA, free fatty acid.

**TABLE 3:** Isolated plant-derived bioactive compounds reported to modulate autophagy in MAFLD models.

Isolated plant-derived bioactive compound	Medicinal plant	Dosage/Route/Time	Autophagy modulator/Positive control	Related target	Pharmacological mechanism	*In vitro*/*In vivo*	Model	Reference
Linarin (C_28_H_32_O_14_)	*Nelumbo nucifera* Gaertn. [Nelumbonaceae; stamen, folium]	10, 20, and 40 μg/mL, medicated culture medium for 6 and 24 h; 10, 20, and 40 mg/kg/day, intragastric administration for 4 weeks	Not mentioned	ACO, CPT1A, PPARα, SREBP-1C, ACC, FAS, TNF-α, IL-6, p-PI3K, PI3K, AKT, p-AKT, p-mTOR, mTOR, LC3B, p62, p-p65, and p65	Modulating the PI3K/AKT/mTOR signaling pathway, autophagy, and gut microbiota	*In vitro* and *in vivo*	OA/PA-induced AML12 cell injury model; HFD-induced C57BL/6 J male mouse model	Lv et al. (2025)
Licochalcone A (C_21_H_22_O_4_)	*Glycyrrhiza uralensis* Fisch. ex DC. [Fabaceae; radix et rhizoma]	0.25, 0.5, 0.75, 1, 2.5, 5, 10, 20, 40, 80, 100, and 150 μM, medicated culture medium for 24 h; 50 and 100 mg/kg/day, intragastric administration for 9 weeks	3BDO and rapamycin	TNF-α, IL-6, IL-13, LC3B, p-mTOR, mTOR, Beclin1, ULK1, p-ULK1, VPS34, ATG5, and ATG12	Inhibition of mTOR expression and regulation of ULK1/Beclin1/VPS34 pathway, thereby promoting autophagy	*In vitro* and *in vivo*	FFA formulated with PA and OA in HepG2 cells; MCD-induced female C57BL/6 mouse model	Li et al. (2025)
Punicalagin (C_48_H_28_O_30_)	*Punica granatum* L. [Lythraceae; pericarpium]	100, 300, and 500 mg/kg/day, intragastric administration for 12 weeks	Nrf2 KO mice	Txinp, NLRP3, ACS, cleaved Caspase1, IL-1β, Nrf2, HO-1, SOD, MDA, GSH, p-p62, Beclin1, LC3B, ATG5/7, p-AMPK, AMPK, p-ULK1, ULK1, mTOR, and p-mTOR	Attenuating oxidative stress and inducing autophagy through the p62/Nrf2 and AMPK/mTOR/ULK1 signaling pathways	*In vivo*	CDAAH diet-induced wild-type or Nrf2 KO mouse model	Ma et al. (2025)
Phillygenin (C_21_H_24_O_6_)	*Reynoutria japonica* Houtt. [Polygonaceae; rhizoma]	10, 20, 30, 40, 50, and 60 μM, medicated culture medium for 24 h; 12.5, 25, and 50 mg/kg/day, intragastric administration for 14 weeks	Simvastatin, chloroquine, and siTFEB	LC3B, p62, ATP6V1A, LAMP1, PGC1α, TFEB, Lamin A, IL-1β, IL-6, TNF-α, and NLRP3	Restoring lipophagy and suppressing lipid accumulation and inflammation by regulating the Ca^2+^–calcineurin–TFEB axis	*In vitro* and *in vivo*	PA-induced AML12 cell injury model; HFD-induced male C57BL/6J mouse model	Zhou et al. (2022)
Resveratrol (C_14_H_12_O_3_)	*Reynoutria japonica* Houtt. [Polygonaceae; rhizoma]	5 and 25 mg/kg/day, intragastric administration for 4 weeks	ULK1 KO mice	AMPK, IκBα, NF-κB, SREBP-1c, TNF-α, and ULK1	Regulating autophagic and IκBα–NF–κB pathways	*In vivo*	HFD-induced wild-type or ULK1 KO mouse model	Li et al. (2014)
Dioscin (C_45_H_72_O_16_)	*Dioscorea oppositifolia* L. [Dioscoreaceae; rhizoma]	20, 40, and 80 mg/kg/day, intragastric administration for 10 weeks	Pyrrolidine dithiocarbamate, and ST2825	HO-1, Nrf2, GSS, KEAP1, SOD-2, AP-1, IKβ-α, CYP2E1, COX-2, NF-κB, HMGB1, p-p38, p38, p-JNK, JNK, p-ERK, ERK, p-p38, p38, p-JNK, JNK, p-ERK, ERK, p-mTOR, mTOR, LC3B, Beclin1, ATG5, ACADM, PPARα, LXRα, ACADS, ACSL1, ACSL5, GPAT, HMGCR, HMGCS1, and SREBP-2	Attenuating oxidative damage, suppressing inflammation, inhibiting triglyceride and cholesterol synthesis, promoting fatty acid β-oxidation, down-regulating MAPK phosphorylation levels, and inducing autophagy	*In vivo*	HFD-induced C57BL/6J and ob/ob mouse model	Liu et al. (2015)
Capsaicin (C_19_H_28_O_8_)	*Capsicum annuum* L. [Solanaceae; fructus]	1 μM, medicated culture medium for 24 h; 15 mg/kg/days, intragastric administration for 24 weeks	TRPV1^−/−^mice and ATG5 siRNA	TRPV1, HSL, p-HSL, CPT1, ATGL, FAS, SREBP-1, PPARα, PPARδ, LXR, Beclin1, LC3B, ATG5, ATG7, and TNF-α	TRPV1-mediated PPARδ-dependent autophagy enhancement	*In vivo* and *in vivo*	PA-induced HepG2 cell injury model; HFD-induced C57BL/6 J wild-type mouse and TRPV1 knockout (TRPV1^−/−^) mouse model	Li et al. (2013b)
S-Allylmercaptocysteine (C_6_H_11_NO_2_S_2_)	*Allium sativum* L. [Amaryllidaceae; bulbus]	200 mg/kg, three times per week, intraperitoneal injection for 8 weeks	Not mentioned	cleaved Caspase3/8, p-p53, Cyto c, Bcl-2, Bcl-XL, Bak1, Bax, Fas, TRAIL, FADD, p-LKB1, p-AMPK, p-PI3K, p-AKT, p-mTOR, vps34, Beclin1, ATG12, LC3B, and p62	Inhibition of apoptosis and enhancing autophagy	*In vivo*	HFD-induced female SD rat model	Xiao et al. (2013)
Buddleoside (C_28_H_32_O_14_)	*Chrysanthemum indicum* L. [Asteraceae; flos]	30 mg/kg/2 days, intraperitoneal injection for 4 weeks	AICAR, metformin, torin1, and chloroquine	p-AMPK, p-mTOR, Tfeb, Val81, Arg83, PRKAB1, RPTOR, TFEB, p62, LC3B, and Beclin1	Targeting the AMPK–TFEB signaling pathway	*In vivo*	HFHC-induced C57BL/6J mouse model	Chen et al. (2025a)
Pterostilbene (C_16_H_16_O_3_)	*Pterocarpus indicus* Willd. [Fabaceae; lignum]	12.5 and 25 μM, medicated culture medium for 24 h; 30, 45, and 60 mg/kg/days, intraperitoneal injection for 24 h	3-Methyladenine	Nrf2, PPARα, HO-1, p-ACC, p-AKT, p-AMPKα, p-AMPKβ1, ATG3, ATG5, ATG7, ATG12, ATG16, Beclin1, LC3B, p-PI3K, SREPB-1c, and p-mTORC	Regulating AMPK/mTOR signaling pathways and autophagy by promoting Nrf2	*In vitro* and *in vivo*	OA/PA-induced HepG2 cell injury model; tyloxapol-induced male C57BL/6 mouse model	Shen et al. (2023)
Schisandrin B (C_23_H_28_O_6_)	*Schisandra chinensis* (Turcz.) Baill. [Schisandraceae; fructus]	12.5, 25, and 50 μM, medicated culture medium for 24 h; 50 mg/kg/days, intragastric administration for 5 weeks	3-Methyladenine	p62, LC3B, p-mTOR, p-p70, p-ULK1, p-AMPK, PI3KC3, Beclin1, ATG7, ATG5, LAMP1, CYP7A1, CPT1A, ACOX1, ACADL, ACADM, HMGCS2, and TFEB	Activation of autophagy through the AMPK/mTOR signaling pathway	*In vitro* and *in vivo*	FFA-induced HepG2 and MPHs cell injury model; HFD-induced male C57BL/6J mouse model	Yan et al. (2022)
Dihydromyricetin (C_15_H_12_O_8_)	*Morella rubra* Lour. [Myricaceae; folium]	5, 10, and 20 μM, medicated culture medium for 24 h; 50, 100, and 200 mg/kg/days, intragastric administration for 6 weeks	Metformin, siAMPK, and PGC-1α-specific siRNA	G6Pase, GLUT2, PEPCK, Beclin1, ATG5, LC3B, p-AMPK, PGC-1α, and PPARα	Regulating AMPK/PGC-1α and PPARα-mediated autophagy pathways	*In vitro* and *in vivo*	PA-induced HepG2 cell injury model; HFD-induced SD rat model	Yang et al. (2024b)
Quercetin (C_15_H_10_O_7_)	*Quercus dentata* Thunb. [Fagaceae; folium]	600 μM, medicated culture medium for 24 h; 20 and 80 mg/kg/days, intragastric administration for 4 weeks	3-Methyladenine and chloroquine	TNF-α, IL-6, IL-1β, ATG5, ATG12, LC3B, PINK1, Parkin, NLRP3, ACS, Caspase-1, IL-18, p-NF-κB p65, p-AMPKα, AMPKα, SIRT1, and p-p65	Promotion of AMPK-mediated hepatic mitophagy	*In vitro* and *in vivo*	OA-induced HepG2 cell injury model; MCD-induced male C57BL/6J mouse model	Cao et al. (2023)
Cyanidin-3-O-glucoside (C_21_H_21_ClO_11_)	*Glycine* max (L.) Merri. [Fabaceae; semen]	100 μM, medicated culture medium for 24 h; 2 g/kg/days, intragastric administration for 12 weeks	PINK1 siRNA and AAV8-shPINK1	NLRP3, Pro-Caspase1, IL-1β, TOM20, COX IV, LC3B, PINK1, and Parkin	Promoting PINK1-mediated mitophagy	*In vitro* and *in vivo*	PA-induced HepG2 and AML-12 cell injury model; HFD-induced male C57BL/6J mouse model	Li et al. (2020)
Ginsenoside Rg1 (C_42_H_72_O_14_)	*Panax ginseng* C.A.Mey. [Araliaceae; radix et rhizoma]	40 μM, medicated culture medium for 24 h; 30 mg/kg/days, intragastric administration for 4 weeks	3-Methyladenine and rapamycin	ATG2B, LC3B, p62, NLRP3, cleaved Caspase1, IL-1β, PTEN, p-AKT, and AKT	Regulating the miR-375–3p/ATG2B/PTEN–AKT axis to mediate autophagy and pyroptosis	*In vitro* and *in vivo*	FFA-induced HepG2 cell injury model; MCD-induced male C57BL/6 mouse model	Chen et al. (2023)

MAFLD, metabolic-associated fatty liver disease; OA, oleic acid; PA, palmitic acid; HFD, high-fat diet; FFA, free fatty acid; MCD, methionine–choline-deficient, CDAAH, choline-deficient, L-amino acid-defined high-fat diet, KO, knockout; HFHC, high-fat and high-cholesterol.

### 6.1 TCM compound preparations delay the progression of MAFLD by targeting autophagic flux

Natural TCM formulas are inherently multi-constituent, multi-target, and multi-pathway interventions, conceptually aligning with the parallel pathogenic drivers of MAFLD, including dysregulated lipid metabolism, oxidative stress, organelle stress, and amplified inflammation. Autophagy, a central mechanism for hepatic energy homeostasis and organelle quality control, has thus emerged as a key mechanistic target for TCM-based interventions. Currently, formulas reported to ameliorate MAFLD by modulating autophagy-related pathways include Zexie Decoction (ZXD), Chaize Mixture (CZM), Shenling Baizhu Powder (SL), modified Suanmei-Tang, Si-Wei-Qing-Gan-Tang, Ger-Gen-Chyn-Lian-Tang, and Jiang Zhi Granule. Among these, ZXD, CZM, and SL are the most representative, supported by both experimental evidence and a relatively well-defined “formula-constituent-pathway” linkage centered on energy sensing and inflammation–autophagy coupling.

At the formula level, ZXD, first documented in the *Jin Gui Yao Lue*, consists of *Alisma plantago-aquatica* L. (15 g) [Alismataceae; rhizoma] and *Atractylodes macrocephala* Koidz. (6 g) [Asteraceae; rhizoma] in a 5:2 ratio. Clinical evidence suggests that ZXD improves liver function indices and circulating lipids in MAFLD, indicating a hepatoprotective signal (Wang M.-Y. et al., 2022). Mechanistically, in methionine–choline-deficient (MCD) diet-induced MAFLD mice, ZXD lowered plasma ALT levels, increased hepatic SOD activity, reduced MDA levels, and decreased α-SMA expression. Autophagy-related assessments showed fewer autophagosomes by TEM and a dose-dependent reduction in LC3-II by immunoblotting (Xu et al., 2019). As these are largely static markers, they support modulation of autophagy-related processes but do not, in isolation, define changes in autophagic flux (Xu et al., 2019). Evidence for ZXD is further supported by studies on its constituent medicinal plants and representative compounds. Extracts of Alisma plantago-aquatica L. improved plasma lipid profiles and attenuated steatosis in HFD-induced MAFLD animal models (Hong et al., 2006) and suppressed lipogenesis in NEFA-treated HepG2 cells (Jeong et al., 2016). Triterpenoid alisol B 23-acetate was reported to prevent murine MAFLD via FXR activation (Meng et al., 2017). Regarding *A. macrocephala* Koidz., anti-inflammatory activity has been documented (Li et al., 2007), and fermented preparations improved lipid metabolism in HFD-fed obese rats (Wang et al., 2015). Collectively, these findings are consistent with a combined lipid-lowering, anti-inflammatory, and antioxidative stress profile that interfaces with autophagy-associated homeostatic regulation.

CZM, derived from ZXD combined with minor Bupleurum decoction, comprises the following medicinal plants: *Bupleurum chinense* DC. (10 g) [Apiaceae; radix], *Scutellaria baicalensis* Georgi (10 g) [Lamiaceae; radix], *Pinellia ternata* (Thunb.) Makino (9 g) [Araceae; rhizoma], *Codonopsis pilosula* (Franch.) Nannf. (12 g) [Campanulaceae; radix], *Glycyrrhiza uralensis* Fisch. ex DC. (5 g) [Fabaceae; radix et rhizoma], *Zingiber officinale* Roscoe (10 g) [Zingiberaceae; rhizoma], *Ziziphus jujuba* Mill. (10 g) [Rhamnaceae; fructus], *A. macrocephala* Koidz. (15 g) [Asteraceae; rhizoma], and *A. plantago-aquatica* L. (10 g) [Alismataceae; rhizoma]. It has been reported to reduce total cholesterol (TC) and total triglycerides (TGs) in hyperlipidemic cohorts (Xie et al., 2014) and is proposed to ameliorate MAFLD through coordinated effects on inflammation, lipid metabolism, oxidative stress, and insulin sensitivity (Shi et al., 2025; Xie et al., 2022; Zou et al., 2019; Hu et al., 2020). At the pathway level, alisol A 23-acetate activates autophagy-associated signaling via the AMPK/mTOR/ULK1 pathway, concomitantly reducing ROS and inflammatory mediator release (Wu et al., 2018). Additionally, saikosaponin A/D ameliorates HFD-induced MAFLD by modulating triglyceride and fatty acid metabolism (Li X. et al., 2021). In MAFLD models, CZM reduced ALT and AST levels, as well as hepatic triglycerides; it downregulated lipogenesis genes (Srebp1 and Fas) and pro-inflammatory cytokines (TNF-α, IL-6, and IL-1β) while increasing Cpt1a expression. CZM also altered the diversity of gut microbiota (Zhang X. et al., 2025). Mechanistically, CZM decreased GLIPR2 expression and modulated autophagy- and inflammasome-associated proteins, including LC3B, p62/SQSTM1, Beclin-1, NLRP3, caspase-1, and IL-1β. These findings support a model in which CZM engages GLIPR2-linked autophagy regulation coupled with suppression of NLRP3 inflammasome activation (Zhang X. et al., 2025).

Beyond the ZXD axis, SL represents a complementary “multi-omics/network” evidence chain. SL is a classic traditional Chinese medicine formula first recorded in the *Tai Ping Hui Min He Ji Ju Fang* (Prescriptions of the Peaceful Benevolent Dispensary) during the Song Dynasty. It is composed of 10 medicinal plants: *Panax ginseng* C.A.Mey. (15 g) [Araliaceae; radix et rhizoma], *Poria cocos* (Schw.) Wolf (15 g) [Polyporaceae; sclerotium], *A. macrocephala* Koidz. (15 g) [Asteraceae; rhizoma], *Dioscorea oppositifolia* L. (15 g) [Dioscoreaceae; rhizoma], *Lablab purpureus* (L.) Sweet (12 g) [Fabaceae; semen], *Nelumbo nucifera* Gaertn. (9 g) [Nelumbonaceae; semen], *Coix lacryma-jobi* var. *ma-yuen* (Rom. Caill.) Stapf (9 g) [Poaceae; semen], *Amomum villosum* Lour. (6 g) [Zingiberaceae; fructus], *Platycodon grandiflorus* (Jacq.) A. DC. (6 g) [Campanulaceae; radix], and *G. uralensis* Fisch. ex DC. (9 g) [Fabaceae; radix et rhizoma]. Clinically, SL is traditionally used to treat spleen deficiency with dampness retention syndrome and is now also applied in managing MAFLD (Pan et al., 2021). It has been associated with anti-inflammatory effects, alterations in hepatic lipid composition, and modulation of SIRT1 signaling (Zhang Y. et al., 2018; Deng et al., 2019). In HFD-induced MAFLD, Pan et al. (2024) integrated phenotyping with autophagy-related assays, targeted energy metabolomics, and network pharmacology, combined with UPLC–MS/MS and experimental validation, to propose a “SL-constituent-target-phenotype” network. SL reduced hepatic lipid accumulation, improved steatosis and IR, and was proposed to mitigate oxidative stress, ER stress, and mitochondrial dysfunction via induction of autophagy-associated protective programs. Through multi-omics integrative analysis, quercetin, tannic acid, kaempferol, formononetin, β-sitosterol, isorhamnetin, and luteolin were identified as the key bioactive compounds in SL. CAT, AKT, eNOS, NQO1, HO-1, and HIF-1α were identified as the critical target genes, while NADP and succinate were identified as the key energy metabolites.

Taken together, ZXD, CZM, and SL, along with their key compounds, consistently improve metabolic and inflammatory phenotypes across clinical observations and experimental models, repeatedly implicating autophagy-related processes (Wang M.-Y. et al., 2022; Xu et al., 2019; Zhang X. et al., 2025; Pan et al., 2024). However, most studies provide limited causal inference regarding autophagy due to incomplete assessment of autophagic flux (e.g., lysosomal inhibition and p62 dynamics) and insufficient genetic or pharmacological perturbation and rescue experiments. Future research should prioritize formula standardization, rigorous quantification of autophagic flux, and pathway-level causality testing (e.g., AMPK/mTOR/ULK1, GLIPR2, NLRP3, FXR, and SIRT1) using harmonized endpoints aligned with contemporary MAFLD definitions.

### 6.2 Medicinal plant extracts delay the progression of MAFLD by targeting autophagic flux

TCM-edible medicinal plants and functional botanical materials play a crucial role in the prevention and management of MAFLD. Preclinical evidence suggests that these materials can improve hepatic steatosis, IR, oxidative stress, and inflammation, with autophagy increasingly recognized as a key regulatory mechanism. Currently, the herbal materials reported to ameliorate MAFLD by modulating autophagy-related processes include *Lycium chinense* Mill., *Cassia obtusifolia* L., *Eucommia ulmoides* Oliv., *Cyclocarya paliurus* (Batalin) Iljinsk., *Morus alba* L., *Annona squamosa* Linn., *Ampelopsis grossedentata* (Hand.-Mazz.) W.T.Wang, *Citrus bergamia* Risso, *Toona sinensis* (A.Juss.) M. Roem., and *Malus pumila* Mill. Among these, *L. chinense* Mill., *C. obtusifolia* L., and *E. ulmoides* Oliv*.* are the most prominent examples as they have been evaluated in diet-induced MAFLD models and are mechanistically linked to autophagy-associated signaling pathways.

*Lycium chinense* Mill. is a widely used medicinal and edible plant whose hepatoprotective effects are primarily attributed to *Lycium barbarum* polysaccharides (LBPs) (Wang J. et al., 2024). LBP is a structurally heterogeneous polysaccharide/proteoglycan fraction with reported antioxidative, anti-inflammatory, and immunomodulatory activities (Tian et al., 2019). Preclinical studies suggest that LBP mitigates CCl_4_-induced acute liver injury and HFD-induced MAFLD (Xiao et al., 2013; Xiao et al., 2014). In an HFD-driven rat MAFLD model, oral administration of LBP (1 mg/kg for 4 weeks) improved IR and glucose homeostasis, reduced serum transaminases, attenuated hepatic lipid accumulation (with lower circulating FFAs), and alleviated fibrosis, oxidative stress, inflammation, and hepatocyte apoptosis. Mechanistically, these benefits were associated with modulation of NF-κB and MAPK signaling pathways, as well as autophagy-related processes (Xiao et al., 2014). In palmitate (PA)-induced hepatocyte steatosis, the authors further proposed that L-arabinose and β-carotene may contribute to LBP’s bioactivity (Xiao et al., 2014).

Cassia seeds (CSs), derived from *C. obtusifolia* L., have traditionally been used to treat metabolic and liver disorders and are associated with reduced hepatic inflammation and oxidative stress (Meng et al., 2019; Yuen et al., 2021). CS extracts may also exert their effects by repairing the gut barrier and remodeling the microbiota (Luo et al., 2021), as well as by enhancing antioxidant defenses and increasing LDLR expression (Luo et al., 2011; Meng et al., 2019). Ding et al. (2023) characterized an aqueous extract of CS using high-performance liquid chromatography (HPLC), identifying compounds such as aurantio-obtusin, rubrofusarin gentiobioside, cassiaside C, emodin, and rhein. They demonstrated that this extract reduced hepatic steatosis and triglyceride (TG) levels in a high-fat, high-sugar-water (HFSW) mouse model and suppressed lipid accumulation in oleic acid and palmitic acid (OA/PA)-treated HepG2 cells. Mechanistically, the anti-steatotic effect was attributed to the inhibition of fatty acid synthase (FASN)-dependent lipogenesis, accompanied by activation of AMPK and autophagic markers such as LC3-II/LC3-I and ATG5.

*Eucommia ulmoides* leaves are used as functional foods and have been studied for their metabolic benefits. Flavonoids and phenolics are considered the primary active compounds relevant to MAFLD (Pisonero-Vaquero et al., 2015). Previous research demonstrated that leaf extracts promote fatty acid oxidation via the PPARα and PPARδ pathways and reduce serum triglycerides in rats fed a high-fructose, high-fat diet (Kobayashi et al., 2012). Using network pharmacology, molecular docking, and resin-enriched fractions, Gong et al. (2022) identified flavonoids and phenolics as key bioactive components. They found that treatment with leaf extracts reduced intracellular lipid accumulation while increasing PPARγ expression and elevating autophagy levels *in vitro*. The autophagy inhibitor chloroquine (CQ) attenuated PPARγ upregulation, supporting an autophagy-dependent mechanism for PPARγ activation.

Collectively, these studies identify autophagy-linked signaling as a convergent mechanism through which LBP, CS aqueous extract, and *E. ulmoides* leaf ameliorate diet-induced hepatic steatosis and MAFLD phenotypes (Xiao et al., 2014; Ding et al., 2023; Gong et al., 2022). However, most evidence relies on static markers; future research should quantify autophagic flux and strengthen causal inferences by employing genetic or pharmacological perturbation and rescue experiments.

### 6.3 Bioactive compounds of natural medicinal plants delay the progression of MAFLD by targeting autophagic flux

MAFLD represents a significant and growing global health challenge, driving the pursuit of safe and effective metabolic therapies derived from natural products. Plant-derived monomers constitute a valuable resource for drug discovery, offering numerous candidates capable of modulating lipid metabolism and inflammation through autophagy-linked signaling pathways. For example, linarin, licochalcone A (Lico A), punicalagin (PUN), phillygenin, resveratrol, dioscin, capsaicin, S-allylmercaptocysteine, buddleoside, pterostilbene, schisandrin B, dihydromyricetin, quercetin, cyanidin-3-O-glucoside, and ginsenoside Rg1 have been reported to improve MAFLD phenotypes by targeting autophagy-related axes such as PI3K/AKT/mTOR, ULK1/Beclin-1/VPS34, and p62/Nrf2-AMPK/mTOR/ULK1. Among these, linarin, Lico A, and PUN exemplify flavonoid-driven autophagy modulation, while punicalagin is a representative polyphenol that combines antioxidant and anti-inflammatory effects with autophagy induction, supported by genetic evidence demonstrating NRF2 dependence *in vivo*.

Linarin is a plant-derived flavonoid reported to exhibit a wide range of bioactivities, including anti-aging properties, anti-stress activity, inhibition of hyperglycemia, bidirectional regulation, anti-fatigue effects, and enhancement of learning and memory (Mottaghipisheh et al., 2021). In MAFLD models, Lv et al. (2025) demonstrated that linarin reduced lipid accumulation in OA/PA-treated AML12 cells and in HFD-fed mice. This effect was accompanied by increased expression of autophagy-related markers and decreased release of inflammatory mediators. Additionally, 16S rRNA sequencing revealed microbiota remodeling, characterized by a reduced Firmicutes/Bacteroidetes ratio and an increased abundance of Akkermansia and Bifidobacterium. Mechanistically, linarin is proposed to activate autophagy by inhibiting the PI3K/AKT/mTOR signaling pathway, thereby alleviating hepatic steatosis and inflammation.

Lico A, a major flavonoid derived from *G. uralensis* Fisch. ex DC., exhibits well-documented anti-inflammatory activity (Olloquequi et al., 2023; Liu et al., 2024). In both an MCD-diet-fed mouse model and an FFA (PA/OA)-induced steatosis model, Lico A reduced hepatocellular lipid droplets and improved liver injury and lipid parameters, including ALT/AST, TG, CHO, HDL-C, and LDL-C. Mechanistically, Lico A downregulated mTOR and upregulated autophagy-related factors such as ULK1, BECN1, VPS34/PIK3C3, ATG5, and ATG12, consistent with activation of the ULK1/Beclin-1/VPS34 axis and enhanced autophagy-related processes (Li et al., 2025).

PUN, a major polyphenol found in the peel of *Punica granatum* L., exhibits antioxidant and anti-inflammatory properties (Lyu et al., 2017). In a choline-deficient, L-amino acid-defined high-fat (CDAAH) diet-induced model of MAFLD, oral administration of PUN (100, 300, or 500 mg/kg/day for 12 weeks) improved liver injury, with the 300 mg/kg/day dose demonstrating maximal efficacy. This improvement was evidenced by reductions in ALT and AST levels, hepatic LDH activity, and histopathological changes (Ma et al., 2025). PUN suppressed hepatic inflammation and inhibited TXNIP/NLRP3 signaling, reducing oxidative stress by lowering hepatic MDA levels and ROS accumulation, while simultaneously enhancing the activities of SOD and glutathione peroxidase. Additionally, PUN induced autophagy through the p62/Nrf2 and AMPK/mTOR/ULK1 signaling pathways (Ma et al., 2025). Notably, the protective effects were significantly diminished in Nrf2-knockout mice, indicating an NRF2-dependent mechanism (Ma et al., 2025).

Collectively, these studies implicate autophagy-linked signaling pathways involving PI3K/AKT/mTOR, ULK1/Beclin-1/VPS34, p62/Nrf2, and AMPK/mTOR/ULK1 as a convergent axis through which linarin, Lico A, and PUN ameliorate the phenotypes of MAFLD (Lv et al., 2025; Li et al., 2025; Ma et al., 2025).

## 7 Perspectives and conclusion

The prevalence of MAFLD is increasing worldwide, driven by an interconnected pathogenic network that includes IR, lipotoxicity, oxidative stress, mitochondrial and ER stress, chronic low-grade inflammation, and gut–liver axis dysfunction. This systems-level complexity limits the ability of single-target agents to provide durable benefits across the disease continuum, from steatosis to fibrosis. Botanical medicines and TCM formulas, characterized by their multi-constituent and multi-pathway pharmacology, therefore represent a plausible complementary strategy. By simultaneously targeting hepatic lipid metabolism (lipogenesis versus FA oxidation), insulin sensitivity, inflammatory amplification, and gut barrier–microbiome homeostasis, these interventions may more effectively address the multifactorial biology of MAFLD. Consistent with their long-standing use in Asian medical systems, medicinal edible plants (e.g., *L. chinense* Miller, *C. obtusifolia* L., and *E. ulmoides* leaves) and classical formulas (e.g., ZXD and SL) have demonstrated preclinical and early clinical evidence of improving steatosis and fibrotic pathology.

Medicinal plants have long served as a vital source for drug development, offering a diverse array of bioactive compounds that provide a rich chemical and biological foundation for treating various diseases. However, despite the promising potential demonstrated by these plant resources in traditional applications and preliminary research, systematically developing them into clinical drugs that meet modern pharmaceutical standards remains a significant challenge. The complexity of bioactive compounds in medicinal plants, variability in preparation processes, and the unpredictability of their absorption, distribution, metabolism, and excretion in the body collectively constitute the primary translational bottlenecks. Factors such as differing geographical origins, harvest times, and processing methods can cause significant fluctuations in the chemical composition of plant materials, thereby affecting batch-to-batch consistency and the reproducibility of therapeutic efficacy. Furthermore, the multi-constituent and multi-target modes of action of herbal medicines make elucidating their mechanisms challenging. Additionally, issues such as low bioavailability, rapid metabolism, and potential drug interactions of certain bioactive compounds limit their safety and efficacy in clinical applications. Therefore, to facilitate the transformation of natural herbal medicines into modern pharmaceuticals, it is essential to establish a comprehensive quality control system encompassing the entire process from raw materials to finished products. Additionally, integrating multidisciplinary technologies to thoroughly analyze their *in vivo* processes and mechanisms of action is crucial. Overcoming the dual challenges of compound heterogeneity and unclear mechanisms requires standardized and regulated research strategies. This approach will effectively enable the transition of these medicines from traditional remedies to evidence-based therapeutics.

Autophagic dysfunction plays a central role in the onset and progression of MAFLD. Moderate activation of autophagy facilitates the clearance of abnormally accumulated LDs and damaged organelles within hepatocytes, serving as a crucial mechanism for maintaining hepatic metabolic homeostasis. However, there exists a distinct “balance threshold” in the regulation of autophagy. Both excessively high and excessively low levels of autophagy can exacerbate hepatocyte injury. Insufficient autophagy impairs lipid clearance and mitochondrial function, whereas excessive or dysregulated autophagy may, in certain contexts, promote cellular self-digestion or even apoptosis, thereby worsening hepatic inflammation and fibrosis. Therefore, intervention strategies targeting autophagy must focus on precise regulation at various stages of the disease. The goals include restoring the integrity of autophagic flux, enhancing the selectivity of metabolic substrates, and coordinating interactions with signaling pathways such as inflammation and apoptosis. Ultimately, these efforts aim to alleviate hepatic steatosis and prevent the progression of fibrosis. Current research in this field faces numerous challenges, including the lack of a systematic elucidation of the mechanisms underlying dynamic changes in autophagy across different pathological stages and the absence of clinically applicable biomarkers capable of real-time monitoring of autophagy status in liver tissue. Future studies should focus on constructing a dynamic regulatory network of autophagy pathways throughout the progression of MAFLD and promote the development of non-invasive methods for quantitatively assessing autophagic activity. This approach will provide crucial support for translating autophagy-targeted therapies from basic research to clinical practice.

Building on this foundation, this review identifies autophagy as a central hub integrating six major drivers of MAFLD, namely, lipid dysmetabolism, inflammation, mitochondrial dysfunction, oxidative stress, gut microbiota dysbiosis, and ferroptosis. A key innovation of our framework is the delineation of bidirectional vicious cycles that perpetuate this pathological network. For example, lipotoxicity not only results from impaired autophagy but also actively suppresses autophagic flux, creating a self-amplifying loop that accelerates disease progression. Notably, we further integrate botanical evidence through a novel three-tiered hierarchy encompassing formulae, extracts, and bioactive compounds. TCM formulas provide clinical experience and evidence of multi-target synergy, plant extracts offer preliminary pharmacological validation and indicate active constituents, and studies on isolated compounds precisely identify specific molecular targets and pathways at the mechanistic level. Together, these three tiers constitute a complete chain of evidence, progressing from the macroscopic to the microscopic and from the holistic to the reductionist, with integrated visualization highlighting their comprehensive, multi-target profile. This framework directly addresses whether targeting this nodal point enables systematic intervention across the interconnected MAFLD network.

The translational gap from bench to bedside in utilizing natural herbal medicines for MAFLD treatment through autophagy modulation remains a significant challenge. Major obstacles include the lack of standardized quality control for botanical extracts, poor bioavailability, unclear pharmacokinetic profiles of bioactive compounds, and insufficient high-quality evidence from randomized controlled trials to confirm efficacy and safety. Furthermore, the inherent complexity of herbal formulas, where synergistic interactions among multiple constituents underpin their therapeutic effects, poses significant challenges for conventional drug evaluation frameworks, which are typically based on paradigms centered around individual plant-derived bioactive compounds. To bridge this translational gap, several strategies should be prioritized. First, integrating systems pharmacology with network biology can elucidate the interactions within medicinal plant-autophagy networks, thereby identifying core bioactive compounds and their combinatorial rules. Second, advanced formulation technologies (such as phytosomes, micelles, or targeted nano-delivery systems) should be employed to enhance hepatic bioavailability and improve the precision of autophagy-modulating metabolites. Third, establishing evidence-based clinical evaluation frameworks, including rigorous, multicenter, placebo-controlled phase II/III trials utilizing validated non-invasive biomarkers (e.g., serum autophagic flux markers and imaging-based lipid dynamics), is imperative. Such initiatives, combined with regulatory adaptations for medicinal plant development, will not only validate the therapeutic credibility of autophagy-targeted herbal interventions but also accelerate their translation into viable adjunct or mainstream therapies for MAFLD.
